# Cichorins D–F: Three New Compounds from *Cichorium intybus* and Their Biological Effects

**DOI:** 10.3390/molecules25184160

**Published:** 2020-09-11

**Authors:** Muhammad Farooq Khan, Fahd A. Nasr, Omar M. Noman, Nouf Abdulaziz Alyhya, Iftikhar Ali, Mohamad Saoud, Robert Rennert, Mthandazo Dube, Wahid Hussain, Ivan R. Green, Omer Ahmed M. Basudan, Riaz Ullah, Shamsa Hilal Anazi, Hidayat Hussain

**Affiliations:** 1 Department of Zoology, College of Science, King Saud University, P.O. Box 2455, Riyadh 11451, Saudi Arabia; fmuhammad@ksu.edu.sa (M.F.K.); 437203455@ksu.edu.sa (N.A.A.); 439204364@student.ksu.edu.sa (S.H.A.); 2 Medicinal, Aromatic and Poisonous Plants Research Center, College of Pharmacy, King Saud University, Riyadh 11451, Saudi Arabia; fnasr@ksu.edu.sa (F.A.N.); onoman@ksu.edu.sa (O.M.N.); rullah@ksu.edu.sa (R.U.); 3 Shandong Analysis and Test Center, Qilu University of Technology (Shandong Academy of Sciences), Jinan 250014, China; iftikharpcr@yahoo.com; 4 Department of Chemistry, Karakoram International University, Gilgit 15100, Pakistan; 5 Department of Bioorganic Chemistry, Leibniz Institute of Plant Biochemistry, Weinberg 3, D-06120 Halle (Salle), Germany; Mohamad.Saoud@ipb-halle.de (M.S.); Robert.Rennert@ipb-halle.de (R.R.); Mthandazo.Dube@ipb-halle.de (M.D.); 6 Department of Botany, Government Post Graduate College Parachinar, Parachinar 26300, District Kurram, Pakistan; wahidhussain@uop.edu.pk; 7 Department of Chemistry and Polymer Science, University of Stellenbosch, Private Bag X1, Matieland, Stellenbosch 7600, South Africa; irg@sun.ac.za; 8 Department of Pharmacognosy, College of Pharmacy, King Saud University, P.O. Box 2455, Riyadh 11451, Saudi Arabia; basudan@ksu.edu.sa

**Keywords:** *Cichorium intybus*, Asteraceae, naphthalene, anthraquinone, natural product, cytotoxic effects

## Abstract

*Cichorium intybus* L., (chicory) is employed in various traditional medicines to treat a wide range of diseases and disorders. In the current investigation, two new naphthalane derivatives viz., cichorins D (**1**) and E (**2**), along with one new anthraquinone cichorin F (**3**), were isolated from *Cichorium intybus.* In addition, three previously reported compounds viz., β-sitosterol (**4**), β-sitosterol β-glucopyranoside (**5**), and stigmasterol (**6**) were also isolated from *Cichorium intybus.* Their structures were established via extensive spectroscopic data, including 1D (^1^H and ^13^C) and 2D NMR (COSY, HSQC and HMBC), and ESIMS. Cichorin E (**2**) has a weak cytotoxic effect on breast cancer cells (MDA-MB-468: IC_50_: 85.9 µM) and Ewing’s sarcoma cells (SK-N-MC: IC_50_: 71.1 µM); cichorin F (**3**) also illustrated weak cytotoxic effects on breast cancer cells (MDA-MB-468: IC_50_: 41.0 µM and MDA-MB-231: IC_50_: 45.6 µM), and SK-N-MC cells (IC_50_: 71.9 µM). Moreover compounds **1**–**3** did not show any promising anthelmintic effects.

## 1. Introduction

**Figure 1:**
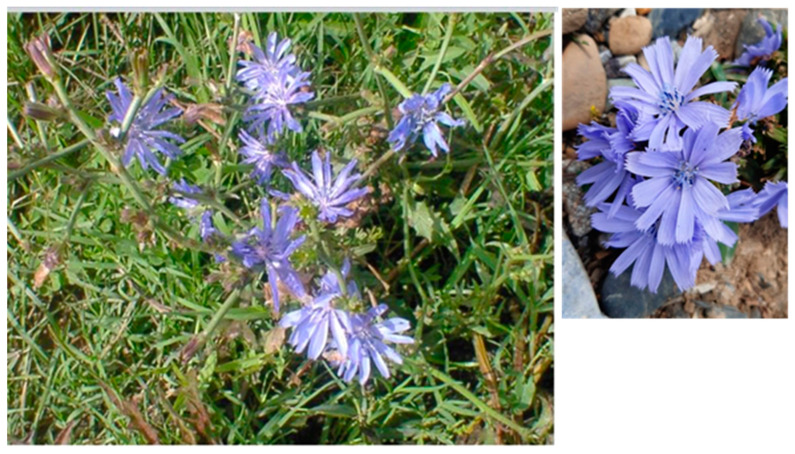
*C. intybus* photographed by the authors.

*Cichorium intybus* L. (chicory) (Figure 1) is a herb of the family Asteraceae. This plant is employed in Uighur folk medicine as a diuretic agent and cholagogic because of its wide range of biological effects viz., antioxidant, anti-inflammatory, and antibacterial [1,2,3]. Additionally, it is also used in traditional medicine to treat diabetes, malaria, gastric ulcers, digestive disorder, and stomachic ailments [4,5]. Moreover, its leaves and roots are reported to be used as an appetizer, digestive, depurative, diuretic, cholagogue, hypoglycemic, and laxative. In addition, a root decoction of *C. intybus* has been employed to treat liver enlargement, jaundice, and gout [4].

Chicory is considered safe to be used in food or in medicine [6]. In addition, the Food and Drug Administration (FDA) has categorized *C. intybus* extract as “generally to be regarded as safe” and included the plant in the “Everything Added to Food in the United States” category [7]. Chicory is used as a tonic in Indian traditional medicine and is commonly used to treat diarrhea and enlarged spleen problems [4]. Moreover, various *C. intybus* extracts have demonstrated a wide range of biological and pharmacological properties viz., anti-hyperuricemia, anti-inflammatory, and antidiabetic, antinematodal, antioxidant and antiproliferative, hepatoprotective, antibacterial, and anti-protozoal effects [4,8,9,10,11,12,13,14,15,16,17,18].

It has been reported that *C. intybus* extracts have a cytotoxic effect on breast cancer (MCF-7) [19], amelanotic melanoma (C32), prostate cancer (LNCaP), renal adenocarcinoma (ACHN) [20], leukemia cells [21], Ehrlich ascites carcinoma [22], prostate cancer (PC-3) [23], breast cells (T47D and SKBR3) [23,24], and cervical cells (HeLa) [25]. Moreover sesquiterpenes reported from *C. intybus* have shown cytotoxic effects on ovarian cancer cells [26], murine lymphoma [27], and leukemia cells [28]. *C. intybus* is a nutritious forb employed to enhance the nutritive value for grazing ruminants [29]. It has been reported that gastrointestinal parasites infected small ruminants after eating (Puna) chicory [29]. Numerous reports have shown direct evidence of the anthelmintic effects of *C. intybus* viz., decreased worm egg numbers in feces [29,30], decreased abomasal worm burdens [29,31], decreased male worms in parasitized animals [29,32], and decreased ability of infective larvae [29,32]. Some reports have demonstrated that sesquiterpene lactones are responsible for anthelmintic effects because these compounds are reported to have significant anthelmintic effects [29,33].

**Figure 2:**
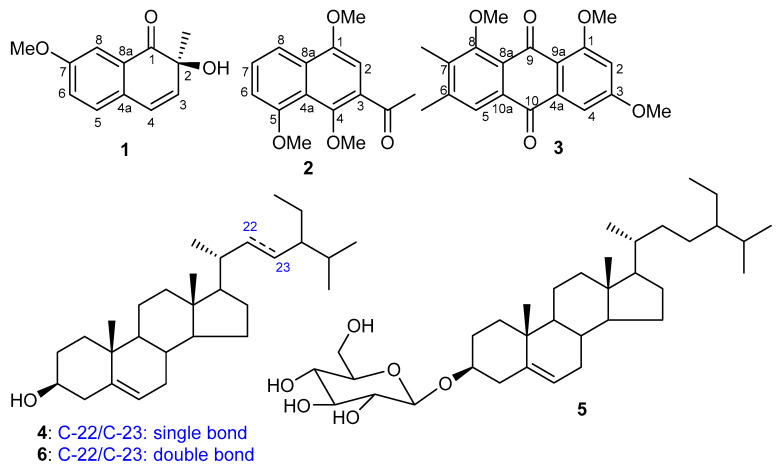
Structures of compounds isolated from *C. intybus*.

The literature has revealed that various natural products with a diverse range of structural skeletons have been reported from *C. intybus* viz., caffeoylquinic acids [34], flavonenes and their glycosides [34], anthocyanins and their glycosides [34], sesquiterpenes [35], steroids [36], triterpenes [37], and benzo-isochromenes [37,38]. In this investigation, we isolated and characterized three new compounds viz., cichorins D–F (**1**–**3**) (Figure 2) along with three reported compounds viz., β-sitosterol (**4**), β-sitosterol β-glucopyranoside (**5**), and stigmasterol (**6**). In the current study, three new compounds **1**–**3** and three known compounds **4**–**6** were isolated from *C. intybus.* Compounds **1**–**3** were evaluated for cytotoxic and anthelmintic effects. 

## 2. Results and Discussion

*C. intybus* was initially extracted with EtOH and ethanolic extract was subjected to repeated column chromatography, providing three new compounds viz., **1**–**3** (Figure 2), along with three known compounds. Cichorin D (**1**) was isolated as a white solid and the IR spectrum demonstrated the presence of a benzene ring (1580 and 1420 cm^−1^), hydroxyl (3390 cm^−1^), and carbonyl group (1630 cm^−1^) (see Experimental section). The molecular formula of compound **1** was established to be C_12_H_12_O_3_ based on the HRESIMS, along with ^1^H and ^13^C NMR spectral analyses. The ^1^H NMR spectrum (Table 1) demonstrated the presence of an ABM spin system of an aromatic ring at δ_H_ 7.62 (d, *J* = 8.0 Hz, 1H, H-5), 6.98 (dd, *J* = 8.0, 2.6 Hz, H-6), and 6.82 (d, *J* = 2.6 Hz, 1H, H-8). The COSY correlations further confirmed that two protons viz., δ_H_ 7.62 (H-5) and 6.98 (H-6) were coupled together through ortho coupling (*J* = 8.0 Hz), while the two protons viz., δ_H_ 6.98 (H-6) and 6.82 (H-8) were coupled to each other via meta coupling (*J* = 2.6 Hz) as well as ortho coupling (*J* = 8.0 Hz).

**Figure 3:**
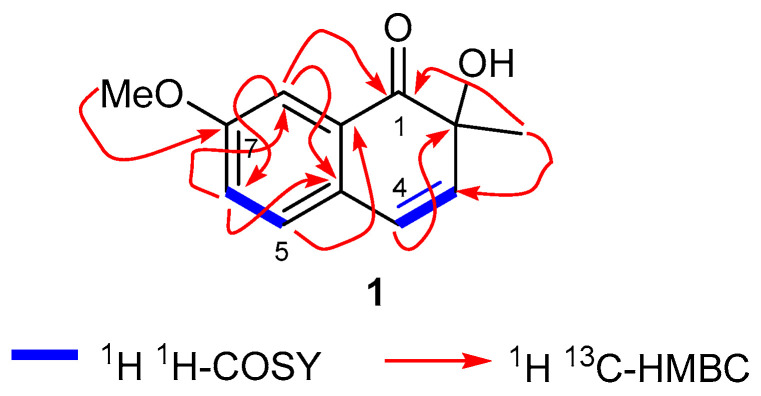
Key COSY and HMBC correlations of cichorin D (**1**).

The ^1^H NMR spectrum also illustrated the presence of an AB olefinic spin system at δ_H_ 7.37 (d, *J* = 9.0 Hz, 1H, H-4) and 6.27 (d, *J* = 9.0 Hz, H-3); this is also evident from COSY correlations (Figure 3). In addition, the ^1^H NMR spectrum revealed the presence of one methoxy group (δ_H_ 3.83, s, 3H, OMe), and one methyl group (δ_H_ 1.52, s, 3H, 2-Me), and this was further confirmed from ^13^C NMR peaks at δ_C_ 55.4 and 33.0, respectively. The ^13^C NMR spectrum along with DEPT experiments illustrated **1** to possess 12 signals attributed to 5 methine, one methyl, one methoxy, and five quaternary carbons. The ^13^C NMR spectrum displayed signals for a ketone at δ_C_ 205.2 along with a saturated quaternary carbon at δ_C_ 76.5 and from these peaks, the proposed structure for this compound is one that possesses a 1,2-dihydronaphthalane skeleton.

The HMBC spectral correlations include the signal at δ_H_ 3.83 to C-7, which established the methoxy group to be at C-7. The regiochemistry at ring A was also confirmed via HMBC correlations (Figure 3) as follows: δ_H_ 6.82 (H-8) to C-6, C-7, C-4a, and C-8a; 6.98 (H-6) to C-4a, C-5, C-7, and C-8; 7.62 (H-5) to C-4a, C-6, and C-7. Moreover the keto group at C-1 was established via HMBC corrections between H-8 (δ_H_ 6.82) to δ_C_ 205.2, along with the correlation of the methyl group (δ_H_ 1.52) to δ_C_ 205.2. In turn, the methyl group at C-2 was evident based on HMBC correlations of this methyl group (δ_H_ 1.52) to C-1, C-2, and C-3. Based on NMR analysis, the compound was found to be a naphthalenone derivative and a literature survey illustrated that the naphthalene analog, berryammone B, reported from the plant *Berrya ammonilla,* also comprises of a keto group at C-1 and a similar C-2 chiral centre as our new compound **1** [39].

Furthermore, the AB olefin spin system protons at δ_H_ 7.37, d, *J* = 9.0 Hz and at 6.27 d, *J* = 9.0 Hz represent H-4 and H-3, respectively, of ring B, which is evident from HMBC correlations depicted in Figure 3. Moreover, the saturated quaternary carbon at δ_C_ 87.1 suggested that the hydroxyl group is attached to C-2. Moreover, the tentative assignment of absolute configuration of compound 1 was established by comparing specific rotation data with a similar published compound, berryammone B [39]. As a result, the chemical structure of compound **1** was confirmed to be 2-hydroxy-7-methoxy-2-methylnaphthalen-1(2*H*)-one, named cichorin D based on the producing plant.

Cichorin E (**2**) was obtained as a light yellow solid and its molecular formula was established to be C_15_H_16_O_4_ via HRESIMS, and 1D and 2D NMR techniques. The IR spectrum showed the presence of a benzene ring (1610 and 1420 cm^−1^) and a carbonyl 1610 (cm^−1^). The ^1^H NMR spectrum (Table 2) illustrates the presence of three methoxy groups (δ_H_ 4.04, 3.99, and 3.82) and a C-acetyl group (2.78 (s, 3H, CO*Me*). Additionally, the ^1^H NMR spectrum (Table 2) illustrated the presence of four aromatic signals at δ_H_ 7.89 (dd, *J* = 1.5, 8.0 Hz, 1H, H-8), 7.51 (t, *J* = 8.0 Hz, 1H, H-7), 7.06 (s, 1H, H-2), 6.98 (dd, *J* = 1.5, 8.0 Hz, H-6). Furthermore COSY correlations, illustrated that the three protons (δ_H_ 7.98, 7.51, and 6.98) were coupled to each other through meta (*J* = 1.5 Hz) and ortho (*J* = 8.0 Hz) coupling. On the other hand, the fourth aromatic proton at δ_H_ 7.06 (s, 1H, H-2) appeared as a singlet. Based on these spectroscopic findings, compound **2** has a naphthalene skeleton bearing a monosubstituted A ring and trisubstituted B ring [40,41,42,43,44].

**Figure 4:**
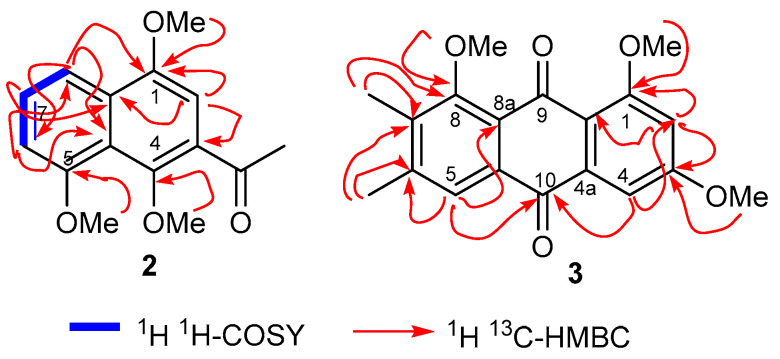
Key COSY and HMBC correlations of cichorin E (**2**) and F (**3**).

The ^13^C NMR and DEPT spectra (see Experimental section) demonstrated that **2** comprised of fifteen carbon signals attributed to four aromatic methane carbon signals, three methoxy, one C-acetyl and its carbonyl, and six quaternary carbons. The regiochemistry of the two methoxy groups and one C-acetyl group in ring A was established from the HMBC correlations: 1-OMe to C-1; H-2 to C-1, C-3, C-4, COMe, and C-8a; COMe to C-3; 4-OMe to C-4. Moreover, HMBC correlations of H-8 to C-8a, C-7, and C-6; H-6 to C-5, C-4a, C-7, C-8, and; H-7 to C-5, C-6, C-8, and C-8a; 5-OMe to C-5 established the ring B regiochemistry (Figure 4). The ^1^H NMR spectral data was similar to that of the synthetic compound 3-acetyl-1,5-dimethoxy-4-naphthol [45], which lacks a methoxy group at C-4. The additional methoxy signal at C-4 i.e., at δ_H_ 3.82 (s, 3H; δ_c_: 63.8) further confirmed the structure of naphthalene analog **2**. Consequently, the cichorin E (**2**) structure was established to be 1-(1,4,8-trimethoxynaphthalen-2-yl) ethan-1-one (**2**).

Cichorin F (**3**) was isolated as a yellow solid, and this molecule has a molecular formula of C_19_H_18_O_5_, as established by the HRESIMS, and ^1^H and ^13^C NMR analysis. The ^1^H NMR spectrum of compound **3** (Table 2) possesses three aromatic protons at δ_H_ 7.81 (s, 1H), 7.34 (d, *J* = 2.0 Hz, 1H), 6.76 (d, *J* = 2.0 Hz), three methoxy groups at δ_H_ 3.97 (s, 3H), 3.95 (s, 3H), 3.92 (s, 3H), and two aromatic methyl groups at δ_H_ 2.39 (s, 3H, Me), and 2.31 (s, 3H, Me). Moreover, the ^13^C NMR spectrum displayed 19 carbon signals, comprising two carbonyls [δ_C_ 181.8 (C-9) and 183.8 (C-10)], nine aromatic quaternary carbons along with three aromatic methine, two methyl, and three methoxy carbons.

These spectroscopic characteristics along with the chemical shifts strongly suggest that compound **3** has a highly substituted anthraquinone skeleton [46], possessing three methoxy carbons at δ_C_ 55.8, 56.5, 61.5 and two methyl groups at δ_C_ 20.7 (6-Me), 12.1 (7-Me). The HMBC spectrum revealed that the proton signals of H-4 (δ_H_ 7.34) and H-5 (δ_H_ 7.81) possess correlations to the C-10 carbonyl moiety at δ_C_ 183.8 (Figure 3) as well as show connectivity to C-4 and C-5, respectively. In addition, the signal at δ_H_ 7.34 (H-4) is meta-coupled (*J* = 2.0 Hz) with the proton signal at δ_H_ 6.76 and thus this signal is assigned to H-2 based on the COSY correlation as well. Furthermore, the H-2 HMBC correlations to C-1, C-3, and C-4 lend further credence to this assignment. In addition, the HMBC correlations of the 1-OMe, 3-OMe and 8-OMe to C-1, C-3 and C-8 demonstrated that these three methoxy groups were located at C-1, C-3, and C-8 respectively. Correlations between the methyl signals at δ_H_ 2.39 (s, 3H, Me) and 2.31 (s, 3H, Me) to C-6 and C-7 demonstrated that these methyl groups were located at C-6 and C-7, respectively. The NMR data of anthraquinone **3** is similar to the natural 8-demethylated anthraquinone, 1-hydroxy-6,8-dimethoxy-2,3-dimethyl-anthraquinone [47], the synthesis of which has been reported [48]. The additional C-8 methoxy signal at δ_H_ 3.92 (s, 3H; δ_c_: 61.5) further confirmed the structure of anthraquinone **3**. The ^1^H,^1^H-COSY, HSQC, and HMBC spectra (Figure 4) established a complete assignment for anthraquinone **3**. As a result, the structure of the new cichorin F (**3**) was determined to be 1,6,8-trimethoxy-2,3-dimethylanthracene-9,10-dione.

The literature revealed that only a few 1,2-dihydronaphthalane derivatives have been reported to bear a keto group at C-1 and a chiral centre at C-2 (similar as compound **1**) having a hydroxyl and alkyl group. Compounds with similar structures include 7-dihydroxy-3(4*H*)-isocadalen-4-one [49], Berryammone B [39], trichbenzoisochromen A [50], and bombamalone A [51]. Moreover, various naphthalene compounds (aglycones and glycosides) have been reported from plants that have similar substitution patterns, as in compound **2**. These compounds are dianellin [52], 5-hydroxydianellin [52], stellalderol [52], parvinaphthols A and B [53], penthosides A and B [54], Eucleanal [40], Eucleanal A and B [41]. Interestingly, anthraquinone **3** has an additional methyl group at C-7 in comparison to trimethoxylated emodin. Moreover few anthraquinones have been reported to have methyl groups at C-6 and C-7 and these compounds are ventinone A [55], 1-hydroxy-6,8-dimethoxy-2,3-dimethylanthraquinone [47], and 1,5-Dihydroxy-8-methoxy-2,3-dimethylanthraquinone [56]. In addition, 2-ethyl-1-hydroxy-8-methoxy-3-methyl anthraquinone and 2-ethyl-1,8-dihydroxy-3-methyl-anthraquinone also have been reported as natural products bearing methyl and ethyl groups at C-6 and C-7 [57]. The three reported natural products viz., β-sitosterol (**4**) [58], β-sitosterol glucopyranoside (**5**) [59], and stigmasterol (**6**) [58] were identified by comparing their NMR data with published data.

## 3. Biological Activities

### 3.1. Cytotoxic Effects

**Figure 5:**
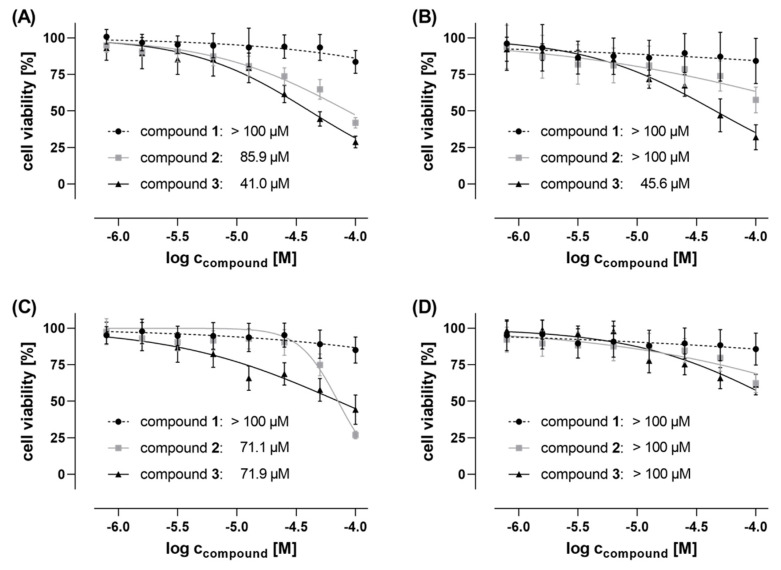
Cell viability of (**A**) breast cancer MDA-MB-468 cells, (**B**) breast cancer MDA-MB-231 cells, (**C**) Ewing’s sarcoma SK-N-MC cells, and (**D**) prostate cancer PC-3 cells treated for 48 h with compounds **1** (●), **2** (■), and **3** (▲), respectively, as determined by MTT assay. Data has been normalized to cell viabilities between 0% (represented by digitonin-treated cells; cytotoxic positive control) and 100% (represented by untreated cells; negative control). IC_50_ values of the anti-proliferative/cytotoxic compound activities have been calculated by using GraphPad Prism 8.0.2 software and are given in micromole concentrations. Data has been collected in two independent biological replicates each with technical quadruplicates.

Compounds **1**, **2** and **3** were screened for cytotoxic effects on the viability of four different human cancer cell lines, namely prostate adenocarcinoma cells (PC-3), triple-negative breast adenocarcinoma (MDA-MB-468 and MDA-MB-231) cells, and Ewing’s sarcoma SK-N-MC cells. Cell viability and cytotoxicity assays were conducted by using tetrazolium salt MTT and crystal violet (CV), respectively, both with colorimetric read-out after 48 h cell treatment. The saponin digitonin (100 µM), was used as a cytotoxic positive control that was subsequently used for the IC_50_ analyses to normalize the raw data to 0% remaining cell viability, while untreated cells (negative control) were used for normalization, complying 100% cell viability. Compounds **1**, **2**, and **3** were tested with concentrations up to 100 µM in order to determine IC_50_ values. Whereas compound **1** was not active (IC_50_s > 100 µM) in all tested cell lines, compound **2** exhibited anti-proliferative effects in the triple-negative breast cancer (TNBC) cell line MDA-MB-468 (IC_50_ = 85.9 µM and 80.0 µM when determined by MTT and CV assay, respectively) and the Ewing’s sarcoma cell line SK-N-MC (IC_50_ = 71.1 µM and 56.4 µM using MTT and CV assay, respectively) (see Figure 5; Figure S1, and Table 3). Contrarily, other tested cell lines, the second TNBC cell line MDA-MB-231 and the prostate cancer PC-3 cells, were detected to be insensitive against compound **2**, with non-detectable IC_50_ values higher than 100 µM. Highest anti-proliferative and cytotoxic activity, respectively, was found for compound **3**. IC_50_ values in the lower micromolar concentration range were detected in both TNBC cell lines, MDA-MB-468, and MDA-MB-231, as well as in Ewing’s sarcoma SK-N-MC cells (41.0 µM, 45.6 µM, and 71.9 µM, respectively, determined by MTT assay; and 47.1 µM, 49.1 µM and 52.9 µM, respectively, determined by CV assay).

In all cases, the IC_50_ values determined by both MTT (assaying the metabolic activity of vital cells) and crystal violet (CV) assay (quantifying the total DNA of the remaining population of viable cells) were in the same concentration range, supporting the validity of these data. Interestingly, the detected anticancer activities are remarkable since both compounds **2** and **3** are much less active in PC-3 cells, a cell line with higher sensitivity against a broader panel of anticancer agents, but show activities (IC_50_s) in the lower micromolar range against breast cancer cell lines MDA-MB-468 and, at least compound **3**, MDA-MB-231, as well as against SK-N-MC cells. This indicates to some extent a cancer cell line selectivity of compounds **2** and **3**, which has to be investigated in more depth in future studies. Moreover, anticancer activities against triple-negative breast cancer (TNBC) cells and Ewing’s sarcoma cells are of particular pharmaceutical interest, since TNBCs and orphan Ewing’s sarcoma still lack satisfactory therapeutic options.

### 3.2. Anthelmintic Effects

*C. intybus* has attracted interest owing to its anthelmintic effects on gastrointestinal (GI) nematodes in monogastrics and ruminants [60]. Scientific evidence has demonstrated that chicory-rich diets can potently decrease infections with GI abomasal nematodes in cattle and sheep in vivo [60]. For this reason, compounds **1**–**3** have been tested for their anthelmintic effects on *Caenorhabditis elegans*. The results showed that compound **1** reduced motility of all worms, even though mortality (10%) was low. On the other hand, compound **2** did not reduce the motility of all worms as **1** did, but it had a higher mortality (24%). Compound **3** had no noticeable effects on worms.

## 4. Conclusions

Phytochemical investigations of *C. intybus* provided two new naphthalane derivatives viz., cichorins D (**1**) and E (**2**) along with one new anthraquinone, cichorin F (**3**). Their structures were established via extensive spectroscopic investigations, including 1D and 2D NMR, and ESIMS techniques. Compound **1** is a 1,2-dihydronapthalane derivative bearing a C-1 keto group; compound **2** is a trimethoxy substituted naphthalene derivative; and cichorin F (**3**) is a highly substituted anthraquinone having three methoxy and two aromatic methyl groups. Cichorin E (**2**) was shown to possess weak cytotoxic effects on MDA-MB-468 and SK-N-MC cancer cells, while cichorin F (**3**) demonstrated weak cytotoxic effects on MDA-MB-468, MDA-MB-231, and SK-N-MC.

## 5. Material and Methods

### 5.1. General Experimental Procedures

IR spectra were recorded using the Nicolet-510P spectrophotometerc (Thermo Fisher Scientific, Waltham, MA, USA); *v*_max_ in cm^−1^. The ^1^H NMR spectra were recorded on Bruker AMX-400 (Bruker Biospin Corp., Billerica, MA, USA) instruments using TMS as an internal reference. The chemical shifts are reported in parts per million (δ) while the coupling constants (*J*) are reported in hertz. The ^13^C NMR spectra were recorded at 100 MHz on the same instrument. Column chromatography (CC) was carried out using silica gel (70–230 and 230–400 mesh; E-Merck, Darmstadt, Germany) and Sephadex LH-20 (Amersham Biosciences AB, Uppsala, Sweden). Aluminum sheets precoated with silica gel 60 F 254 (0.2 mm thick; E-Merck) were used for TLC to check the purity of the compounds and were visualized under UV light (254 and 366 nm), followed by ceric sulfate as the spray reagent.

### 5.2. Plant Material

Whole plant material of *C. intybus* was collected from Parachinar, KPK, Pakistan, in July 2017, and identified by Dr Wahid Hussain (plant taxonomist) at the Department of Botany, Post Graduate College, Parachinar, Pakistan. A voucher specimen (No. B.MF Khan 20. GPGC PCR) has been deposited at the herbarium of the Botany Department, Post Graduate College, Parachinar, Pakistan.

### 5.3. Extraction and Isolation

The air-dried whole plant amterials (3 kg) of *C. intybus* was extracted with EtOH at room temperature. The whole ethanolic extract was evaporated to dryness, yielding 20 g of residue. The whole extract was subjected to Column Chromatography (CC) (silica gel, *n*-hexane, *n*-hexane–EtOAc and EtOAc) yielding 7 fractions (F1-7). Fraction F4 (230 mg) was rechromatographed and eluted with a mixture of *n*-hexane–EtOAc (1.5:8.5) providing cichorins D (**1**; 3.0 mg) and E (**2**; 5.0 mg). Fraction F3 (98 mg) was rechromatographed and eluted with *n*-hexane–EtOAc (7.5:2.5) to afford cichorin F (**3**; 5.4 mg) and β-sitosterol β-glucopyranoside (**5**; 11.3 mg). On the other hand, β-sitosterol (**4**) and stigmasterol (**6**) were purified from fraction F2 by rechromatographing this fraction and eluting with *n*-hexane–EtOAc (1.2:8.8).

#### 5.3.1. Cichorin D (**1**)

While solid; [α]_D_^25^ = +11.2 (*c* 0.26, CH_2_Cl_2_); IR (KBr) *v*_max_: 3410, 2950, 1600, 1420, 1000 cm^−1^; For ^1^H ^13^C NMR: see Table 1; ESI-MS (*m*/*z*): 227.2 [M + Na]^+^, C_12_H_12_NaO_3_; HRESIMS: *m*/*z* 205.0862 [M + H]: (calcd for C_12_H_13_O_3_, 205.0865).

#### 5.3.2. Cichorin E (**2**)

Slightly Yellow gummy solid; IR (KBr) *v*_max_: 1610, 1580, 1425, 1000 cm^−1^; For ^1^H ^13^C NMR: see Table 2; ESI-MS (*m*/*z*): 283.1 [M + Na]^+^, C_15_H_16_NaO_4_; HRESIMS: *m*/*z* 261.1137 [M + H]: (calcd for C_15_H_17_O_4_, 261.1127).

#### 5.3.3. Cichorin F (**3**)

Yellow solid; IR (KBr) *v*_max_: 1600, 1420, 1000 cm^−1^; For ^1^H ^13^C NMR: see Table 2; ESI-MS (*m*/*z*): 349.2 [M + Na]^+^, C_19_H_18_NaO_5_; HRESIMS: *m*/*z* 327.1238 [M + H]: (calcd for C_19_H_19_O_5_, 327.1232).

### 5.4. Cell Culture

Four human cancer cell lines were used to investigate the cytotoxicity and anticancer activity of compounds **1**–**3**. All cell lines were purchased from ATCC (Manassas, VA, USA). The prostate adenocarcinoma cell line PC-3 and triple-negative breast adenocarcinoma (TNBC) cell line MDA-MB-231 were cultured in RPMI 1640 supplemented with 2 mM L-glutamine and 10% heat-inactivated FCS. DMEM supplemented with 2 mM L-glutamine and 10% heat-inactivated FCS was used for the triple-negative breast adenocarcinoma cell line MDA-MB-468 and the Ewing’s sarcoma cell line SK-N-MC. The cells were routinely cultured in T-75 culture flasks in a humidified atmosphere with 5% CO_2_ at 37 °C to reach subconfluency (~70–80%) prior to subsequent usage or subculturing. The adherent cells were rinsed with PBS and detached by using trypsin/EDTA (0.05% in PBS) prior to cell passaging and seeding. Basal cell culture media, FCS, l-glutamine, PBS and trypsin/EDTA for cell culturing were purchased from Capricorn Scientific GmbH (Ebsdorfergrund, Germany). The culture flasks, multi-well plates, and further cell culture plastics were purchased from TPP (Trasadingen, Switzerland) and Greiner Bio-One GmbH (Frickenhausen, Germany), respectively.

#### Cytotoxic Activity—In Vitro Cell Viability Assays

Anti-proliferative and cytotoxic effects of the compounds were investigated by performing colorimetric MTT (3-(4,5-dimethylthiazol-2-yl)-2,5-diphenyltetrazolium bromide) and CV (crystal violet)-based cell viability assays (Sigma-Aldrich, Taufkirchen, Germany), respectively [61,62]. For this purpose, cells were seeded in low densities in 96-well plates (6000 cells/100 µL/well for both PC3 and MDA-MB-468; 4000 cells/100 µL/well for MDA-MB-231; and 12,000 cells/100 µL/well for SK-N-MC) using the aforementioned cell culture media. The cells were allowed to adhere for 24 h, followed by the 48 h compound treatment. Based on 20 mM DMSO stock solutions, the compounds were serially diluted in standard growth media to reach final concentrations of 100, 50, 25, 12.5, 6.25, 3.125, 1.56, and 0.78 µM for cell treatment. For control measures, cells were treated in parallel with 100 µM digitonin (positive control, for data normalization set to 0% cell viability). Each data point was determined in technical quadruplicates and two independent biological replicates. As soon as the 48 h incubation was finished, cell viability was measured.

For the MTT assay, cells were washed once with PBS, followed by incubation with MTT working solution (0.5 mg/mL MTT in culture medium) for 1 h under standard growth conditions. After discarding the MTT solution, DMSO was added in order to dissolve the formed formazan, followed by measuring formazan absorbance at 570 nm, and additionally at the reference/background wavelength of 670 nm, by using a SpectraMax M5 multi-well plate reader (Molecular Devices, San Jose, CA, USA).

For the CV assay, cells were washed once with PBS and fixed with 4% paraformaldehyde (PFA) for 20 min at room temperature (RT). After discarding the PFA solution, the cells were left to dry for 10 min and then stained with 1% crystal violet solution for 15 min at RT. The cells were washed with water and were dried overnight at RT. Afterwards, acetic acid (33% in aqua bidest) was added to the stained cells and absorbance was measured at 570 nm and 670 nm (reference wavelength) using a SpectraMax M5 multi-well plate reader (Molecular Devices, San Jose, CA, USA). For data analyses, GraphPad Prism version 8.0.2 and Microsoft Excel 2013 were used.

### 5.5. Anthelmintic Activity

Anthelmintic activity was performed according to a previously published procedure [63].

## Figures and Tables

**Table 1:** ^1^H NMR (400 MHz) and ^13^C NMR (100 MHz) data of cichorin D (**1**) in CDCl_3_.

No	^1^H NMR	^13^C NMR
1	-	205.2
2	-	76.5
3	6.27 (d, *J* = 9.0 Hz, 1H)	122.9
4	7.37 (d, *J* = 9.0 Hz, 1H)	145.7
4a		129.3
5	7.62 (d, *J* = 8.0 Hz, 1H)	126.8
6	6.98 (dd, *J* = 8.5, 2.6 Hz, 1H)	115.8
7	-	159.1
8	6.82 (d, *J* = 2.6 Hz, 1H)	114.6
8a	-	136.9
7-OMe	3.83 (s, 3H)	55.4
2-Me	1.52 (s, 3H)	33.0
2-OH	3.61 (s, OH, 1H)	

**Table 2:** ^1^H NMR (400 MHz) and ^13^C NMR (100 MHz) data of cichorins E (**2**) and F (**3**) in CDCl_3_.

	Compound 2		Compound 3	
No	^1^H NMR	^13^C NMR	^1^H NMR	^13^C NMR
1	–	151.3		161.7
2	7.06 (s, 1H)	103.2	6.76 (d, *J* = 2.0 Hz, 1H)	105.0
3	–	129.0		163.8
4	–	152.0	7.34 (d, *J* = 2.0 Hz, 1H)	101.9
4a	–	120.1		136.6
5	–	156.8	7.81 (s, 1H)	123.7
6	6.98 (dd, *J* = 1.5, 8.0 Hz, 1H)	107.3		143.7
7	7.51 (t, *J* = 8.0 Hz, 1H)	128.0		139.6
8	7.89 (dd, *J* = 1.5, 8.0 Hz, 1H)	114.8		158.1
8a	–	131.1		131.5
9	–	–		181.8
9a	–	–		118.1
10	–	–		183.8
10a	–	–		125.6
1-OMe	3.99 (s, 3H)	55.7	3.97 (s, 3H)	56.5
3-OMe	–	–	3.95 (s, 3H)	55.8
4-OMe	3.82 (s, 3H)	63.8		
5-Ome	4.04 (s, 3H)	56.1		
8-OMe	–	–	3.92 (s, 3H)	61.5
6-Me	–	–	2.39 (s, 3H)	20.7
7-Me	–	–	2.31 (s, 3H)	12.1
CO*Me*	2.78 (s, 3H)	31.4		
COMe	–	201.2		

**Table 3:** IC_50_ values of the anti-proliferative and cytotoxic effects of compounds **1**, **2**, and **3** as determined after 48 h treatment of breast cancer MDA-MB-468 and MDA-MB-231 cells, Ewing’s sarcoma SK-N-MC cells, and prostate cancer PC-3 cells by using MTT and CV (crystal violet) assays, respectively.

IC_50_ Values (after 48 h Treatment)	Assay	Compound 1	Compound 2	Compound 3
MDA-MB-468 (breast cancer)	MTT	>100 µM	85.9 µM	41.0 µM
	CV	>100 µM	80.0 µM	47.7 µM
MDA-MB-231 (breast cancer)	MTT	>100 µM	>100 µM	45.6 µM
	CV	>100 µM	>100 µM	49.1 µM
SK-N-MC (Ewing’s sarcoma)	MTT	>100 µM	71.1 µM	71.9 µM
	CV	>100 µM	56.4 µM	52.9 µM
PC-3 (prostate cancer)	MTT	>100 µM	>100 µM	>100 µM
	CV	>100 µM	>100 µM	>100 µM

## Supplementary Materials

The following are available online: copies of ^1^H, ^13^C and 2D NMR spectra of compounds **1**–**3**.
