# Supplementary material for: Cichorins D–F: Three New Compounds from Cichorium intybus and Their Biological Effects
Source: Molecules. 2020 Sep 11;25(18):4160. doi: 10.3390/molecules25184160 (PMC7570803; doi:10.3390/molecules25184160)
Supplement: Supplementary file 1 [file molecules-25-04160-s001.pdf]

Supplementary Information

Cichorins D-F: Three new compounds from *Cichorium intybus and their biological effects*

Muhammad Farooq khan[1], Fahd A. Nasr[2], Omar M. Noman[2], Nouf Abdulaziz Alyhya[1], Iftikhar Ali[3,4], Mohamad Saoud[5], Robert Rennert[5], Mthandazo Dube[5], Wahid Hussain[6], Ivan R. Green[7], Omer Ahmed M. Basudan[8], Riaz Ullah[2], Shamsa Hilal Anazi[1], and Hidayat Hussain[*5]

1. Department of Zoology, College of Science, King Saud University, P.O. Box 2455, Riyadh 11451, Saudi Arabia; fmuhammad@ksu.edu.sa; 437203455@ksu.edu.sa; 439204364@student.ksu.edu.sa
2. Medicinal, Aromatic and Poisonous Plants Research Center, College of Pharmacy, King Saud University,Riyadh 11451, Saudi Arabia; fnasr@ksu.edu.sa; onoman@ksu.edu.sa; rullah@ksu.edu.sa
3. Shandong Analysis and Test Center, Qilu University of Technology (Shandong Academy of Sciences), Jinan 250014, China; iftikharpcr@yahoo.com
4. Department of Chemistry, Karakoram International University, Gilgit 15100, Pakistan
5. Department of Bioorganic Chemistry, Leibniz Institute of Plant Biochemistry, Weinberg 3, D-06120 Halle (Salle), Germany; Mohamad.Saoud@ipb-halle.de; Robert.Rennert@ipb-halle.de; Mthandazo.Dube@ipb-halle.de
6. Department of Botany, Government Post Graduate College Parachinar 26300, District Kurram, Pakistan; wahidhussain@uop.edu.pk
7. Department of Chemistry and Polymer Science, University of Stellenbosch, Private Bag X1, Matieland, Stellenbosch 7600, South Africa; irg@sun.ac.za
8. Department of Pharmacognosy, College of Pharmacy, King Saud University, P.O. Box 2455, Riyadh 11451, Saudi Arabia; basudan@ksu.edu.sa

* Correspondence: hussainchem3@gmail.com/Hidayat.Hussain@ipb-halle.de (H.H.); orcid.org/0000-0002-8654-8127

Contents

Page S2: Figure S1. Cell viability of tested cell lines determined by crystal violet (CV) assay.
Page S3: Figure S2. ^{1}H NMR spectrum of cichorin D (**1**) (400 MHz, $CDCl_3$).

Page S4: Figure S3. ^{13}C NMR spectrum of cichorin D (**1**) (400 MHz, $CDCl_3$).

Page S5: Figure S4. DEPT spectrum of cichorin D (**1**) (400 MHz, $CDCl_3$).

Page S6: Figure S5. COSY spectrum of cichorin D (**1**) (400 MHz, $CDCl_3$).

Page S7: Figure S6. HSQC spectrum of cichorin D (**1**) (400 MHz, $CDCl_3$).

Page S8: Figure S7. HMBC spectrum of cichorin D (**1**) (400 MHz, $CDCl_3$).

Page S9: Figure S8. ESIMS spectrum of cichorin D (**1**).

Page S10: Figure S9. HRESIMS spectrum of cichorin D (**1**).

Page S11: Figure S10. ^{1}H NMR spectrum of cichorin E (**2**) (400 MHz, $CDCl_3$).

Page S12: Figure S11. ^{13}C NMR spectrum of cichorin E (**2**) (400 MHz, $CDCl_3$).

Page S13: Figure S12. COSY spectrum of cichorin E (**2**) (400 MHz, $CDCl_3$).

Page S14: Figure S13. HSQC spectrum of cichorin E (**2**) (400 MHz, $CDCl_3$).

Page S15: Figure S14. HSQC spectrum of cichorin E (**2**) (400 MHz, $CDCl_3$).

Page S16: Figure S15. ESIMS spectrum of cichorin E (**2**).

Page S17: Figure S16. HRESIMS spectrum of cichorin E (**2**).

Page S18: Figure S17. ^{1}H NMR spectrum of cichorin F (**3**) (400 MHz, $CDCl_3$).

Page S19: Figure S18. ^{13}C NMR spectrum of cichorin F (**3**) (400 MHz, $CDCl_3$).

Page S20: Figure S19. DEPT spectrum of cichorin F (**3**) (400 MHz, $CDCl_3$).

Page S21: Figure S20. COSY spectrum of cichorin F (**3**) (400 MHz, $CDCl_3$).

Page S22: Figure S21. HSQC spectrum of cichorin F (**3**) (400 MHz, $CDCl_3$).

Page S23: Figure S22. HMBC spectrum of cichorin F (**3**) (400 MHz, $CDCl_3$).

Page S24: Figure S23. ESIMS spectrum of cichorin F (**3**).

Page S25: Figure S24. ESIMS spectrum of cichorin F (**3**).

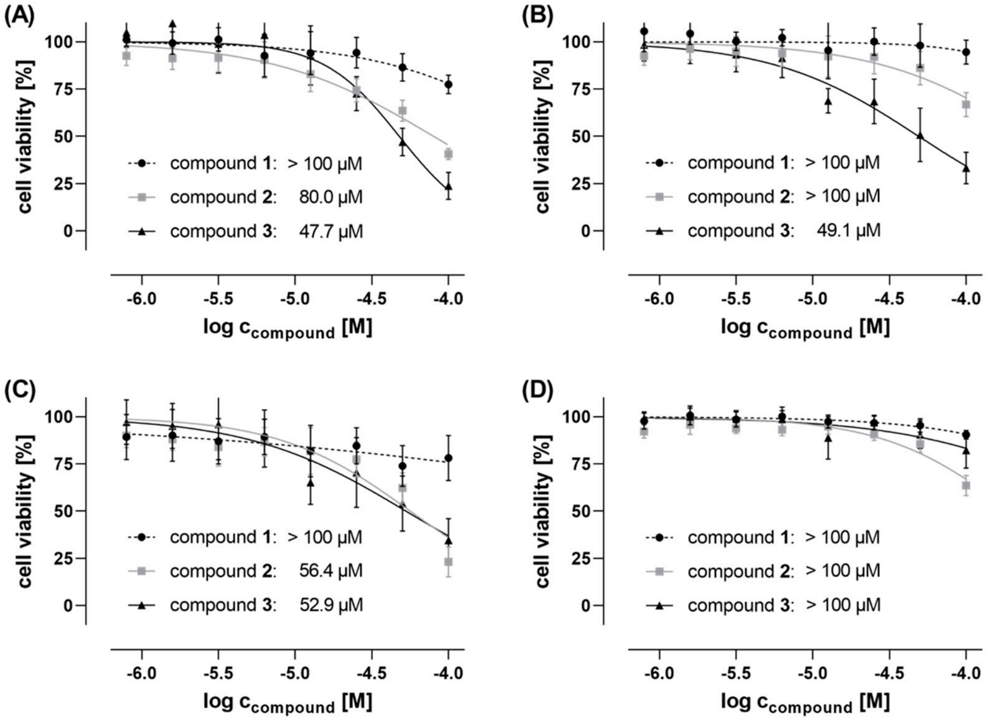

Figure S1. Cell viability of (A) breast cancer MDA-MB-468 cells, (B) breast cancer MDA-MB-231 cells, (C) Ewing's sarcoma SK-N-MC cells, and (D) prostate cancer PC-3 cells treated for 48 h with compounds **1** (●), **2** (■), and **3** (▲), respectively, as determined by crystal violet (CV) assay.

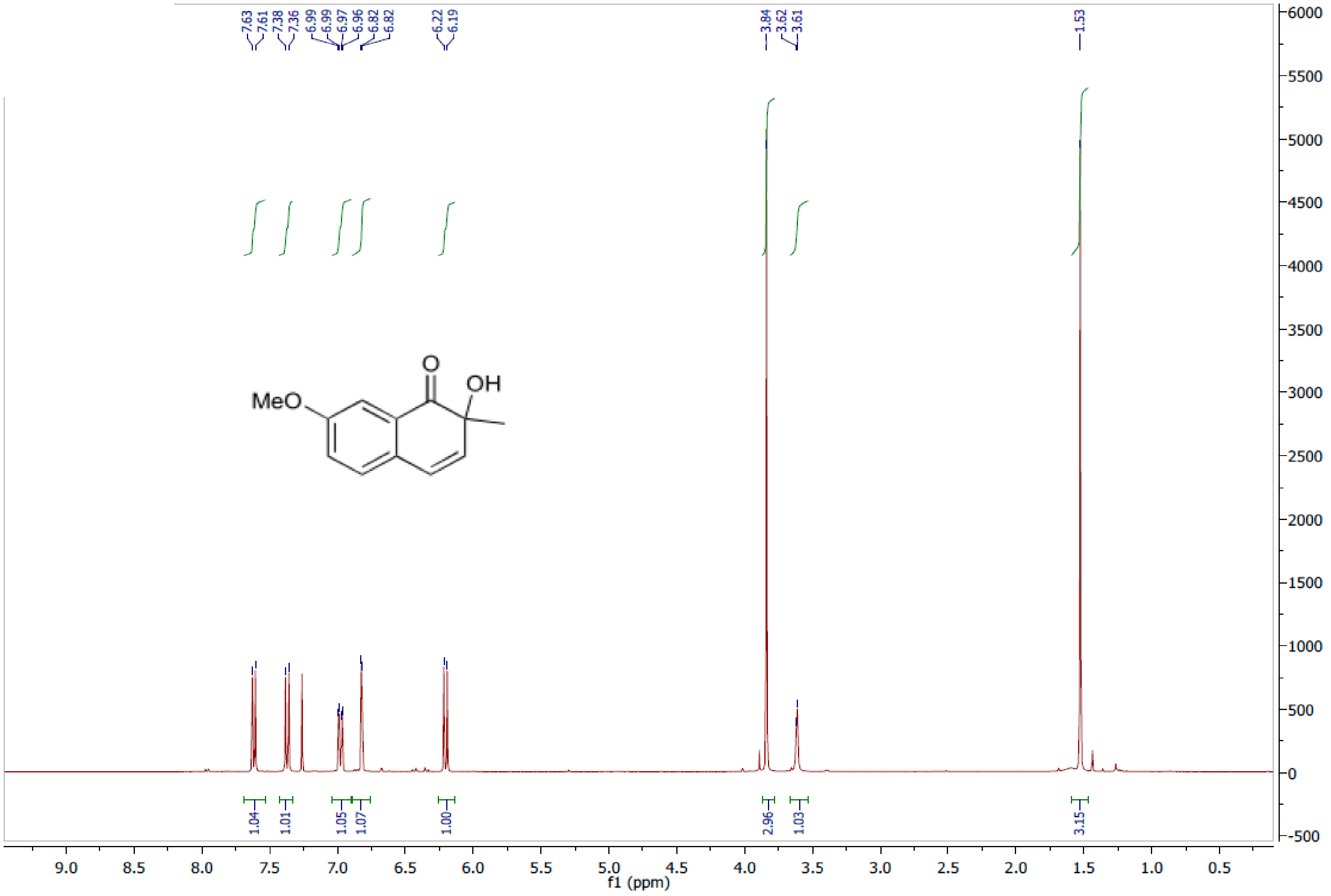

Page S3: Figure S2. ^{1}H NMR spectrum of cichorin D (**1**) (400 MHz, $CDCl_3$).

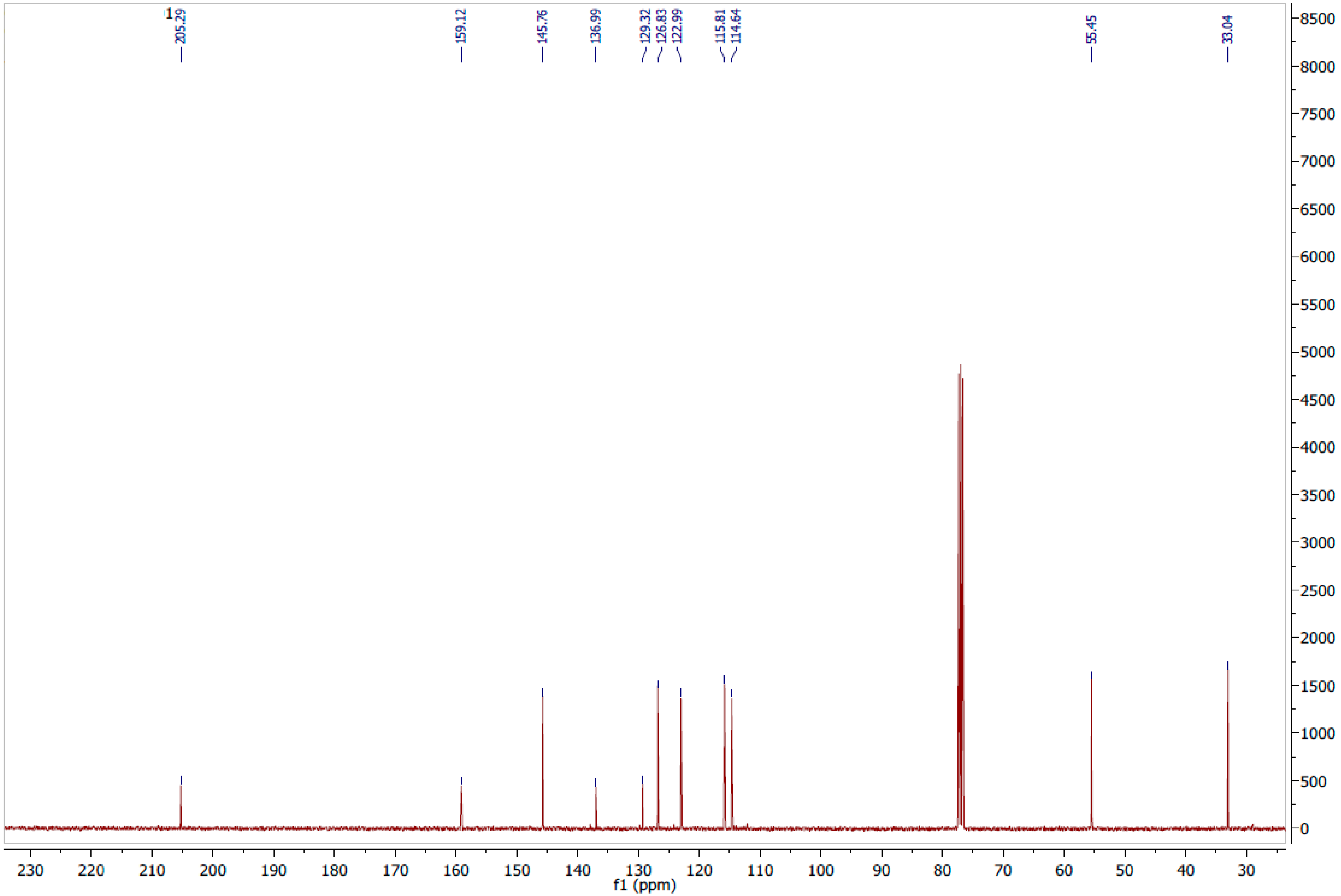

Page S4: Figure S3. ^{13}C NMR spectrum of cichorin D (**1**) (400 MHz, $CDCl_3$).

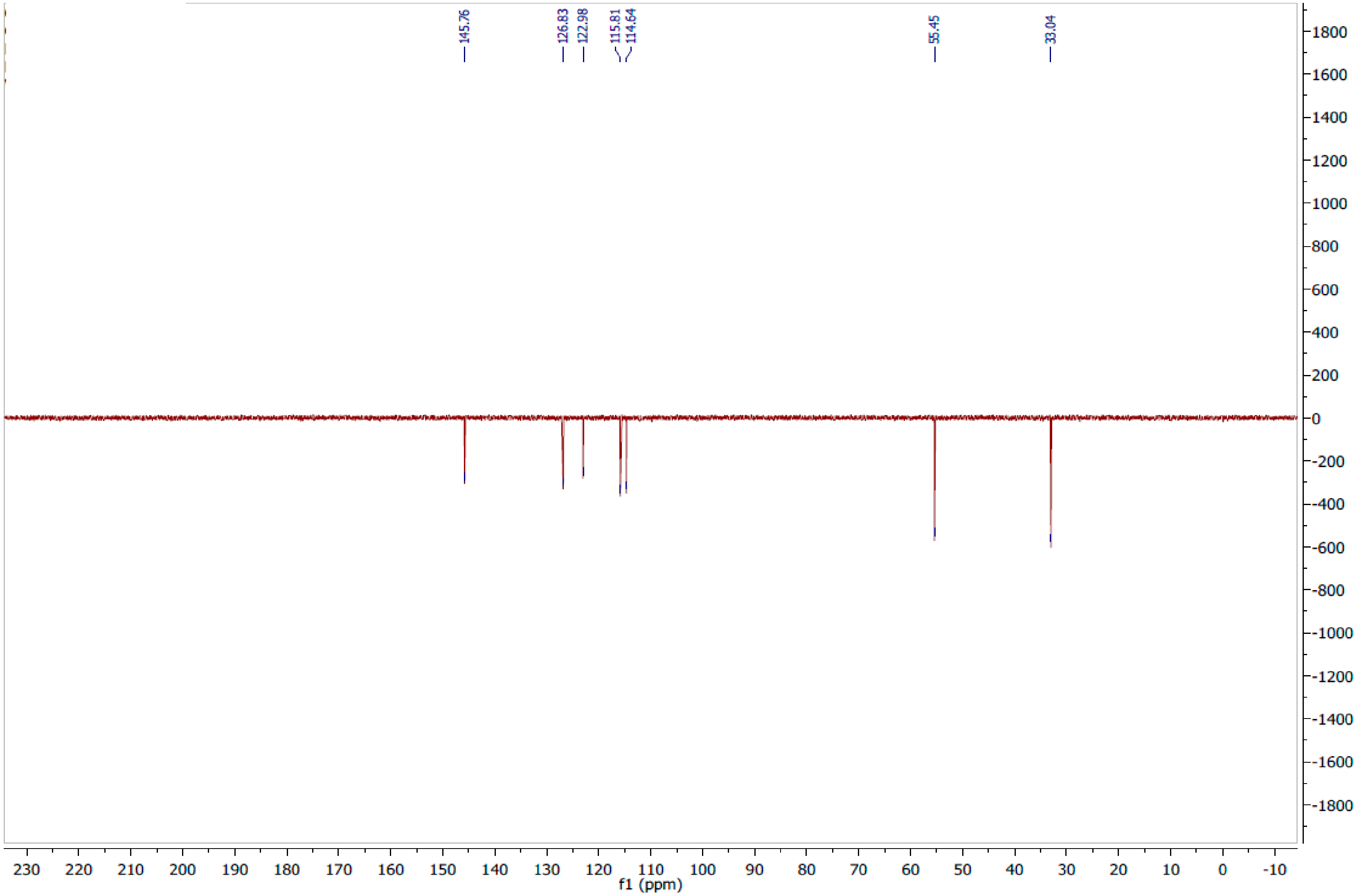

Page S5: Figure S4. DEPT spectrum of cichorin D (**1**) (400 MHz, $CDCl_3$).

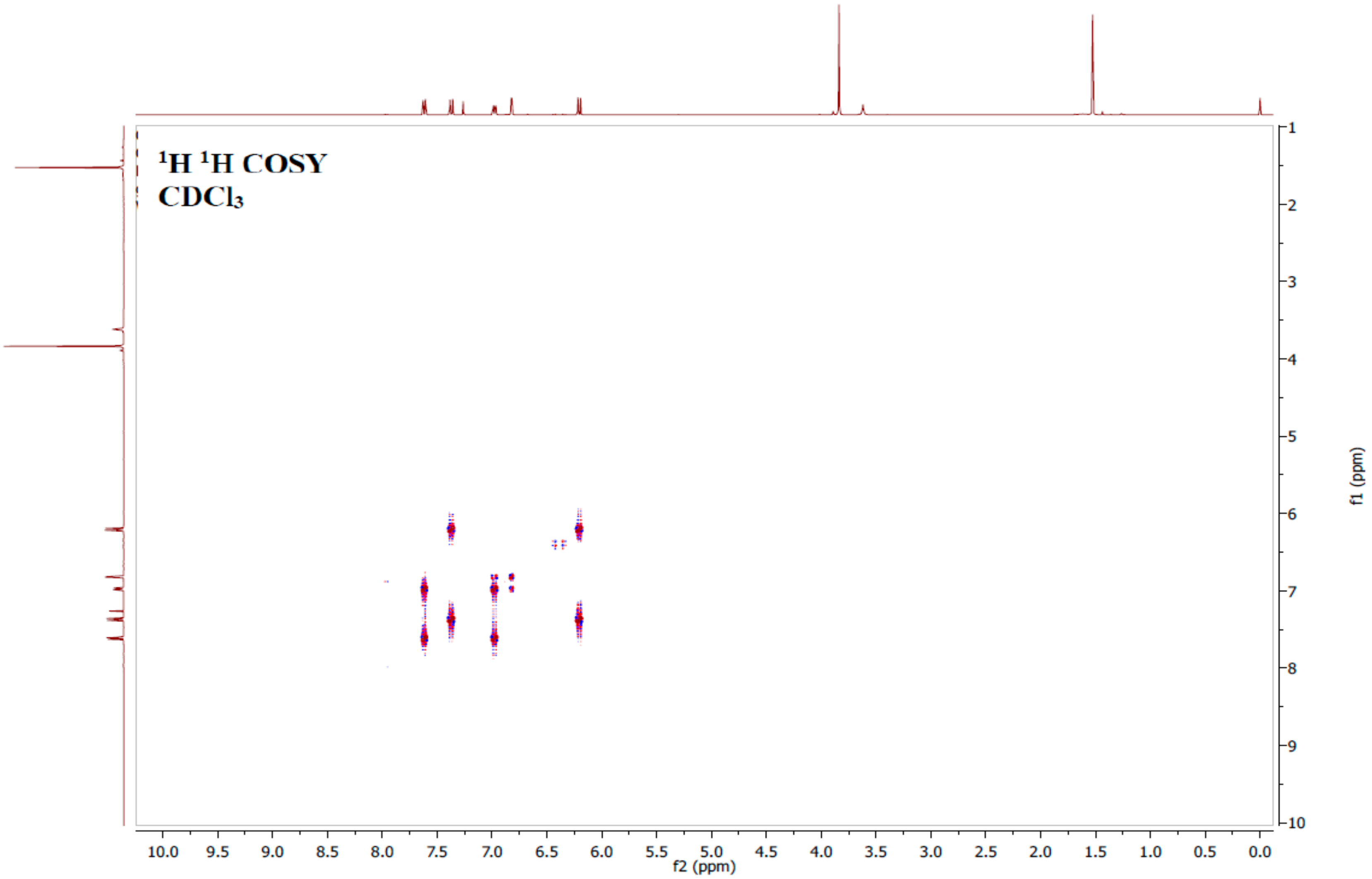

Page S6: Figure S5. COSY spectrum of cichorin D (**1**) (400 MHz, $CDCl_3$).

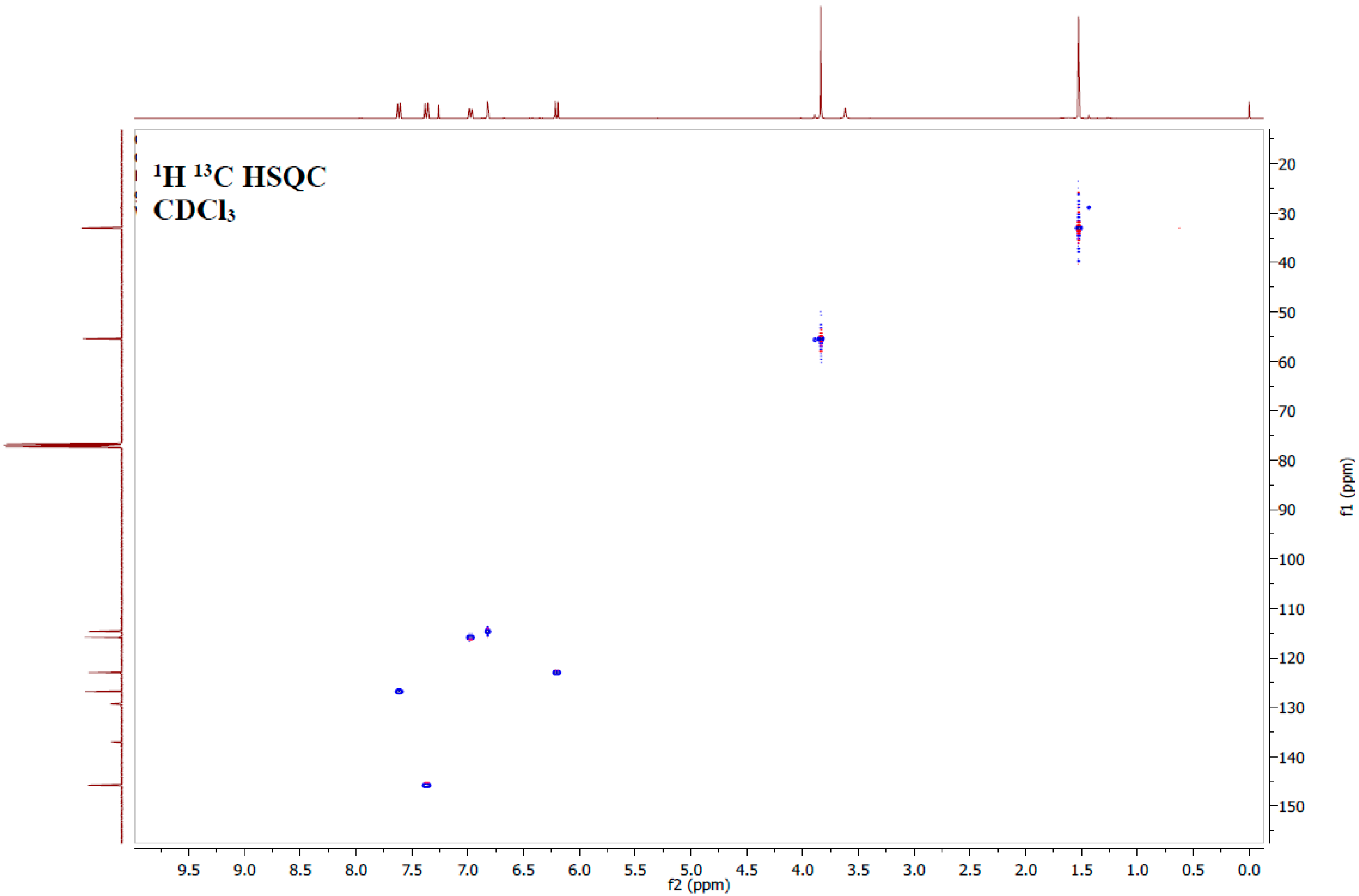

Page S7: Figure S6. HSQC spectrum of cichorin D (**1**) (400 MHz, $CDCl_3$).

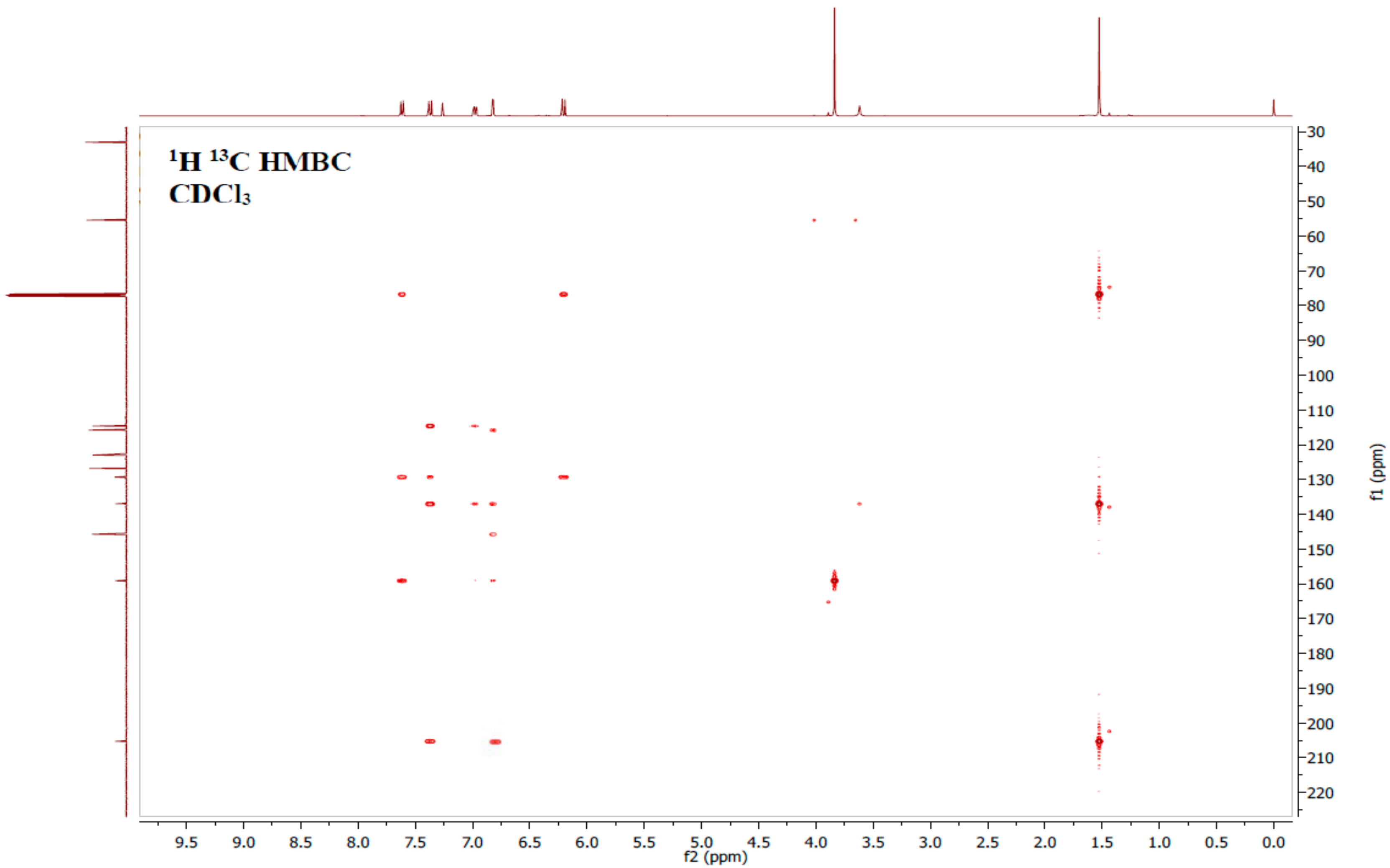

Page S8: Figure S7. HMBC spectrum of cichorin D (**1**) (400 MHz, $CDCl_3$).

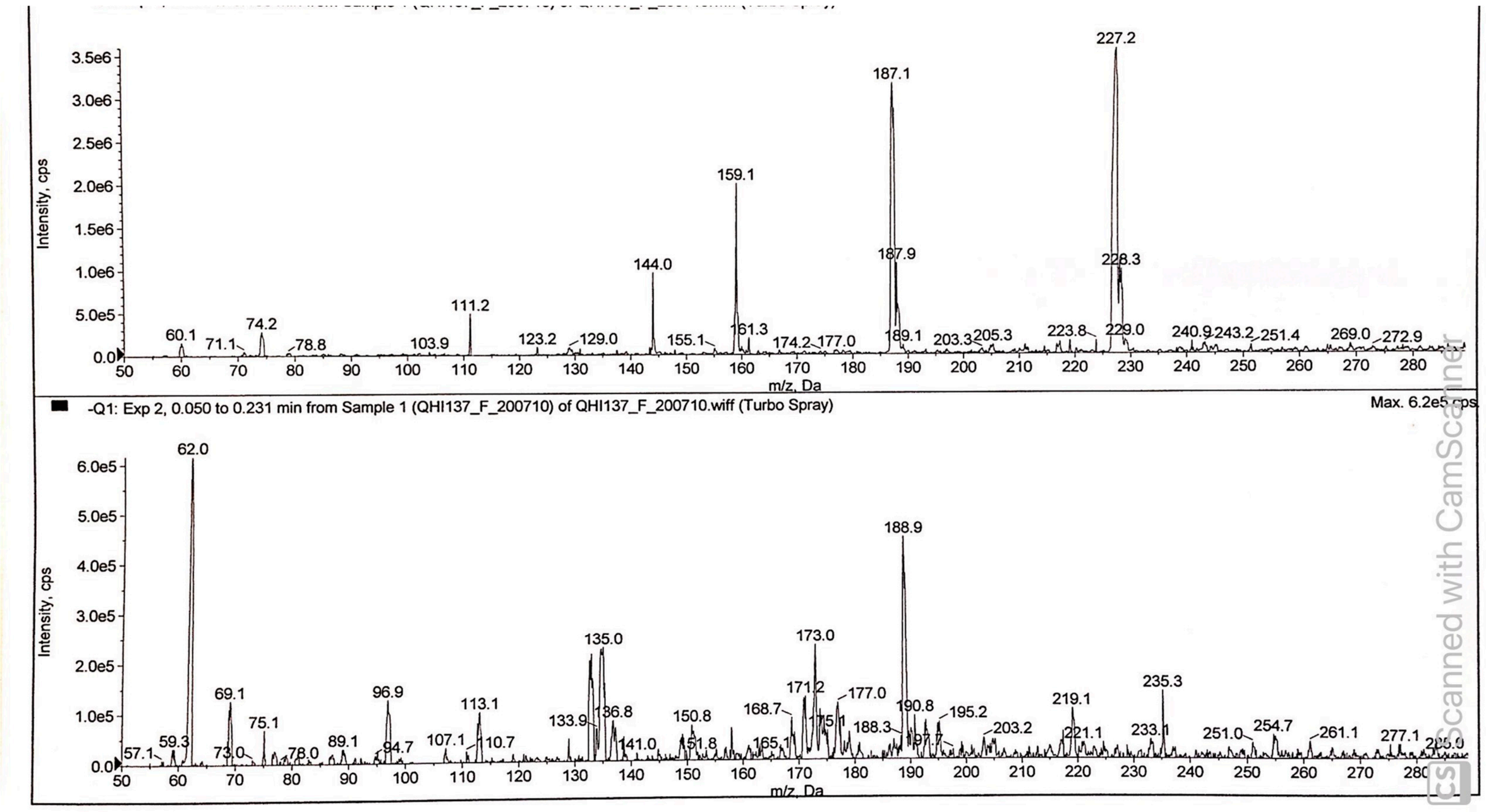

Page S9: Figure S8. ESIMS spectrum of cichorin D (**1**).

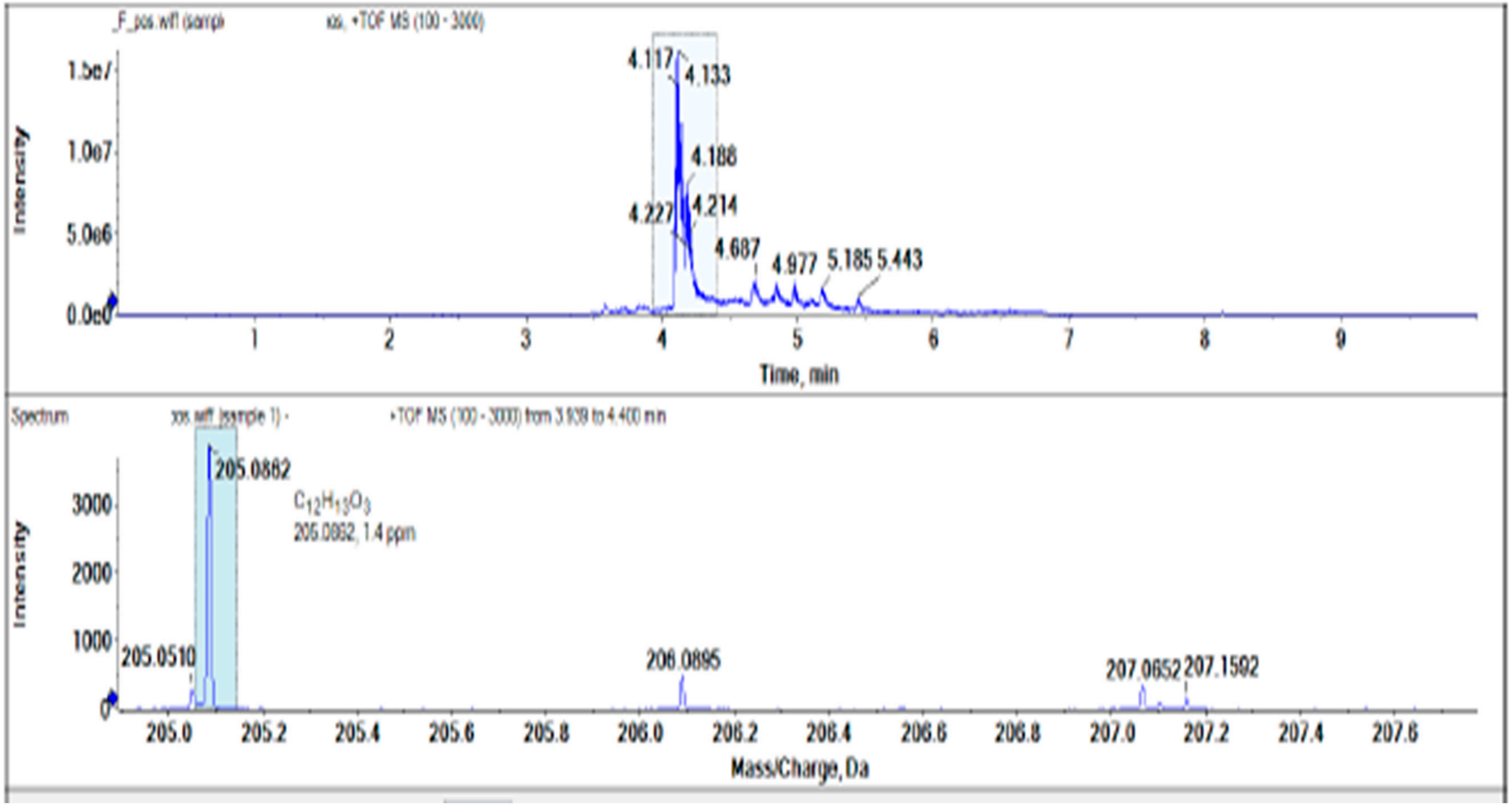

Page S10: Figure S9. HRESIMS spectrum of cichorin D (**1**).

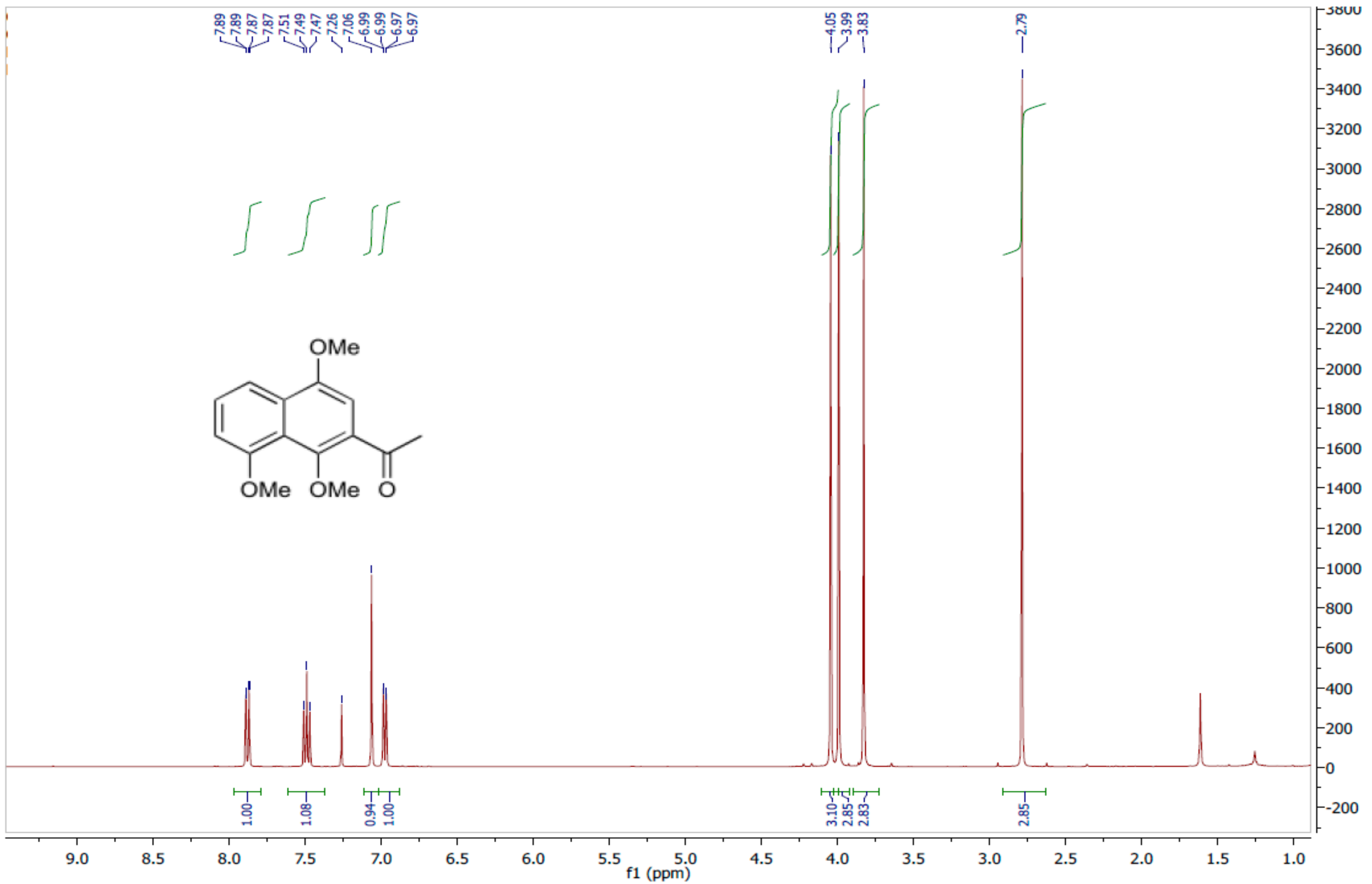

Page S11: Figure S10. ^{1}H NMR spectrum of cichorin E (**2**) (400 MHz, $CDCl_3$).

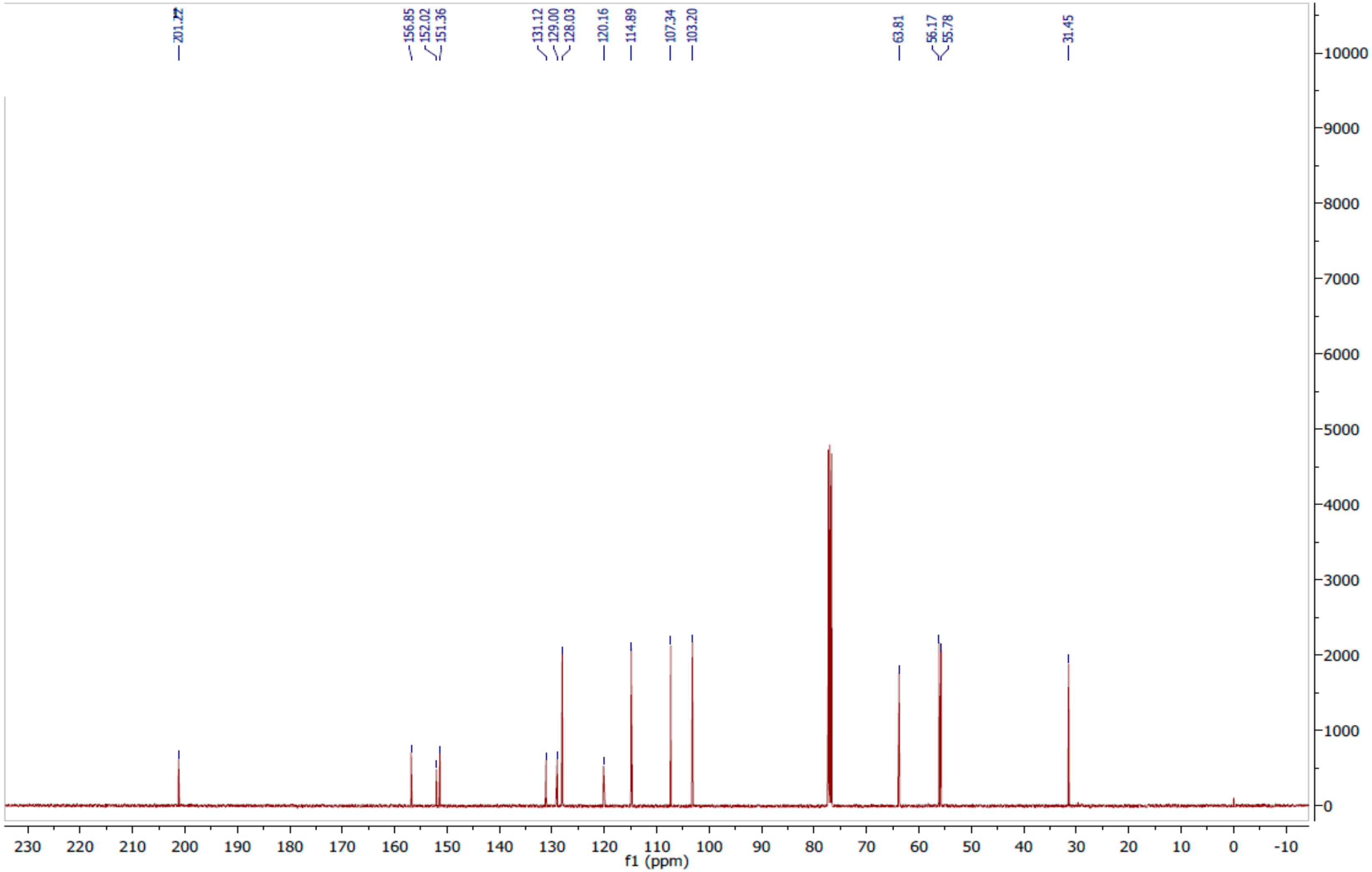

Page S12: Figure S11. ^{13}C NMR spectrum of cichorin E (**2**) (400 MHz, $CDCl_3$).

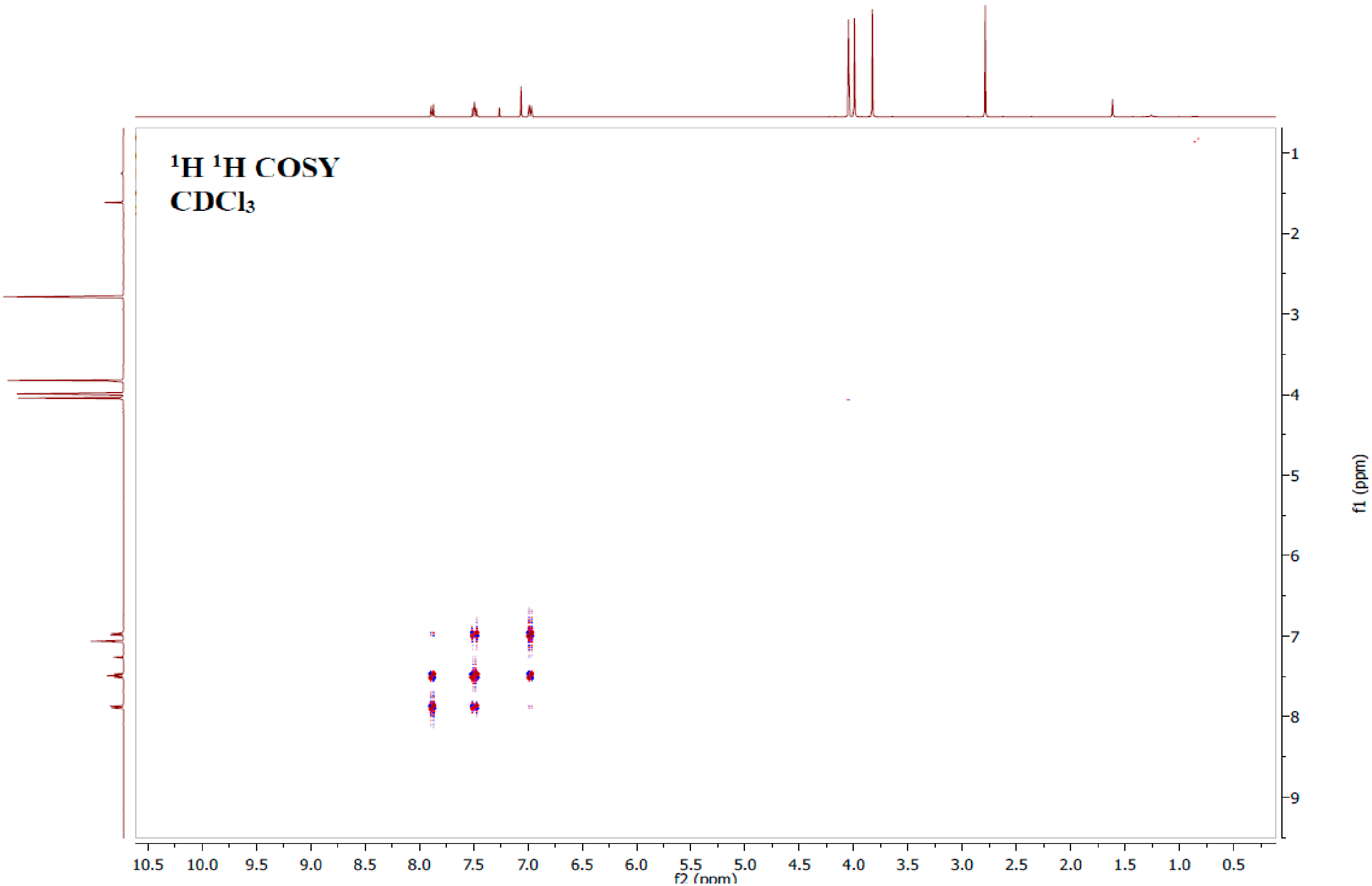

Page S13: Figure S12. COSY spectrum of cichorin E (**2**) (400 MHz, $CDCl_3$).

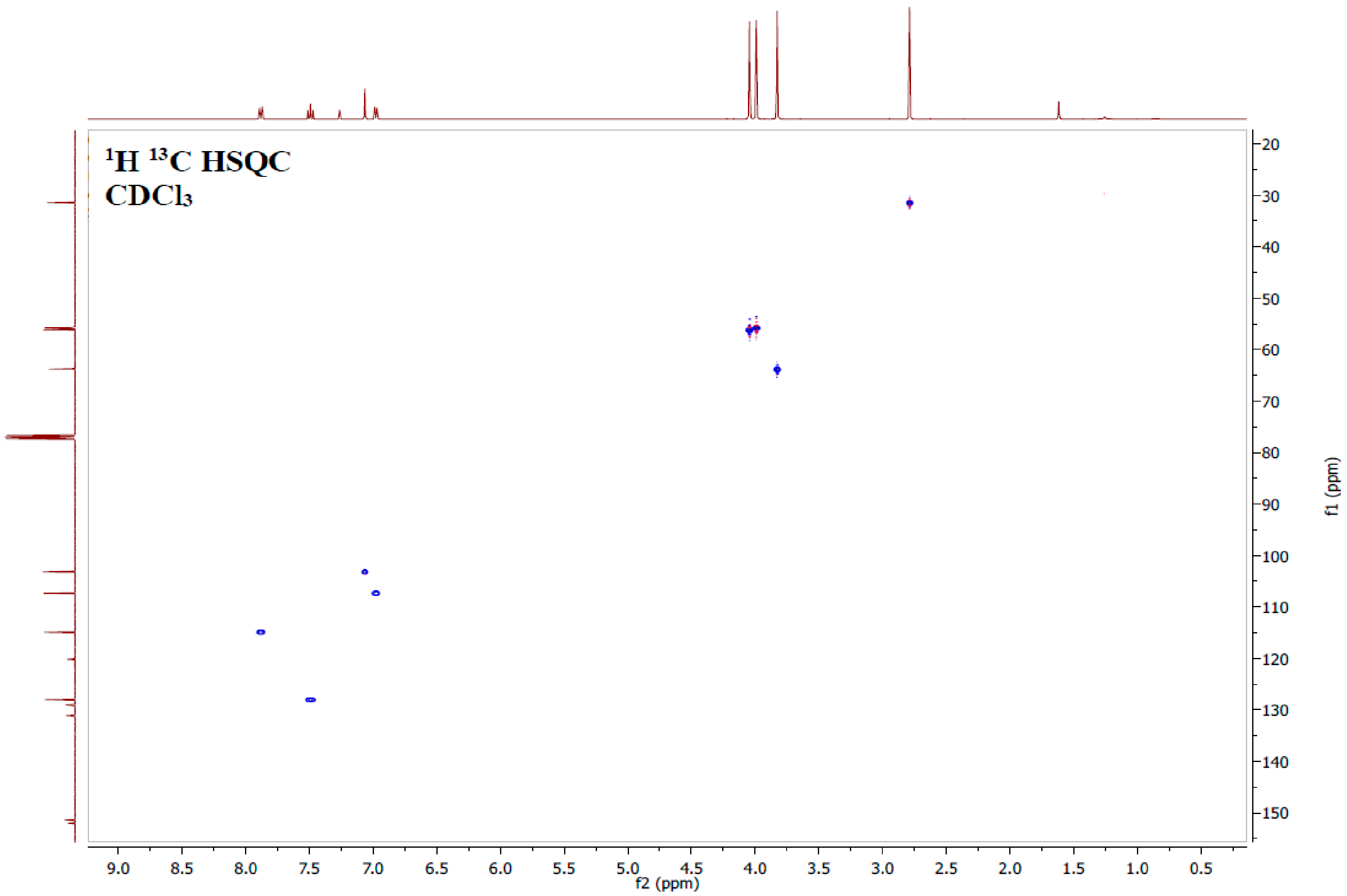

Page S14: Figure S13. HSQC spectrum of cichorin E (**2**) (400 MHz, $CDCl_3$).

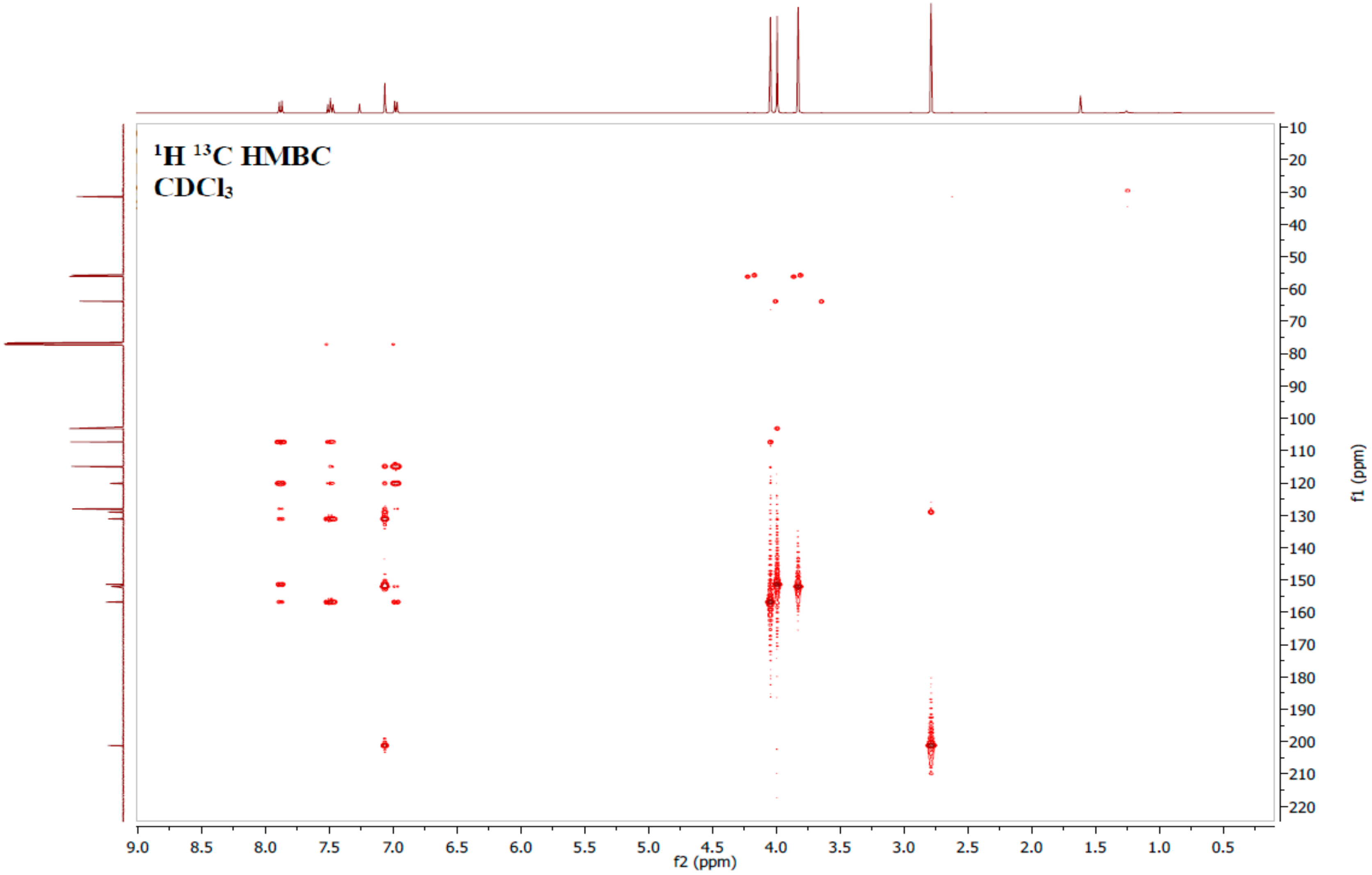

Page S15: Figure S14. HSQC spectrum of cichorin E (**2**) (400 MHz, $CDCl_3$).

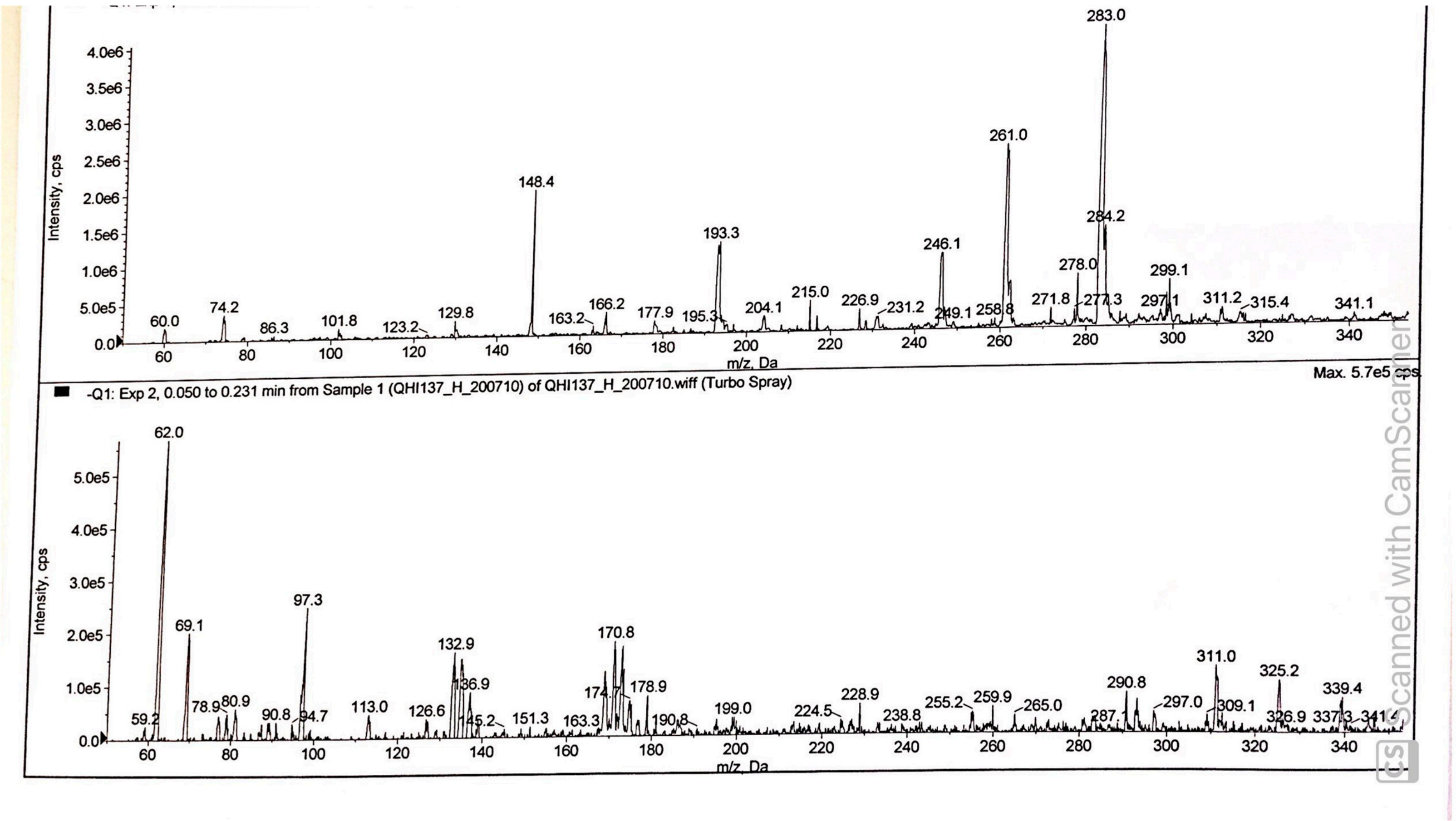

Page S16: Figure S15. ESIMS spectrum of cichorin E (**2**).

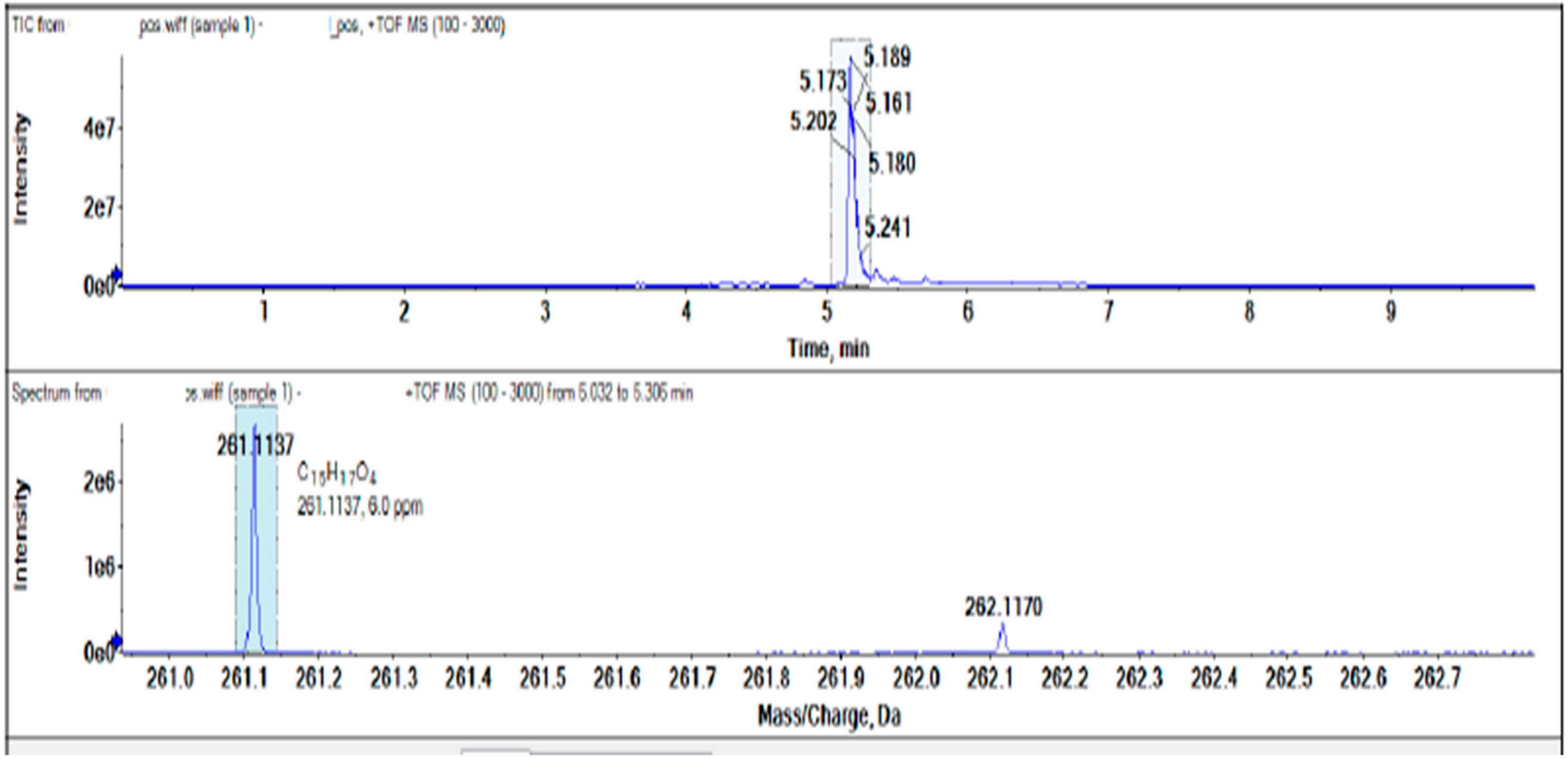

Page S17: Figure S16. HRESIMS spectrum of cichorin E (**2**).

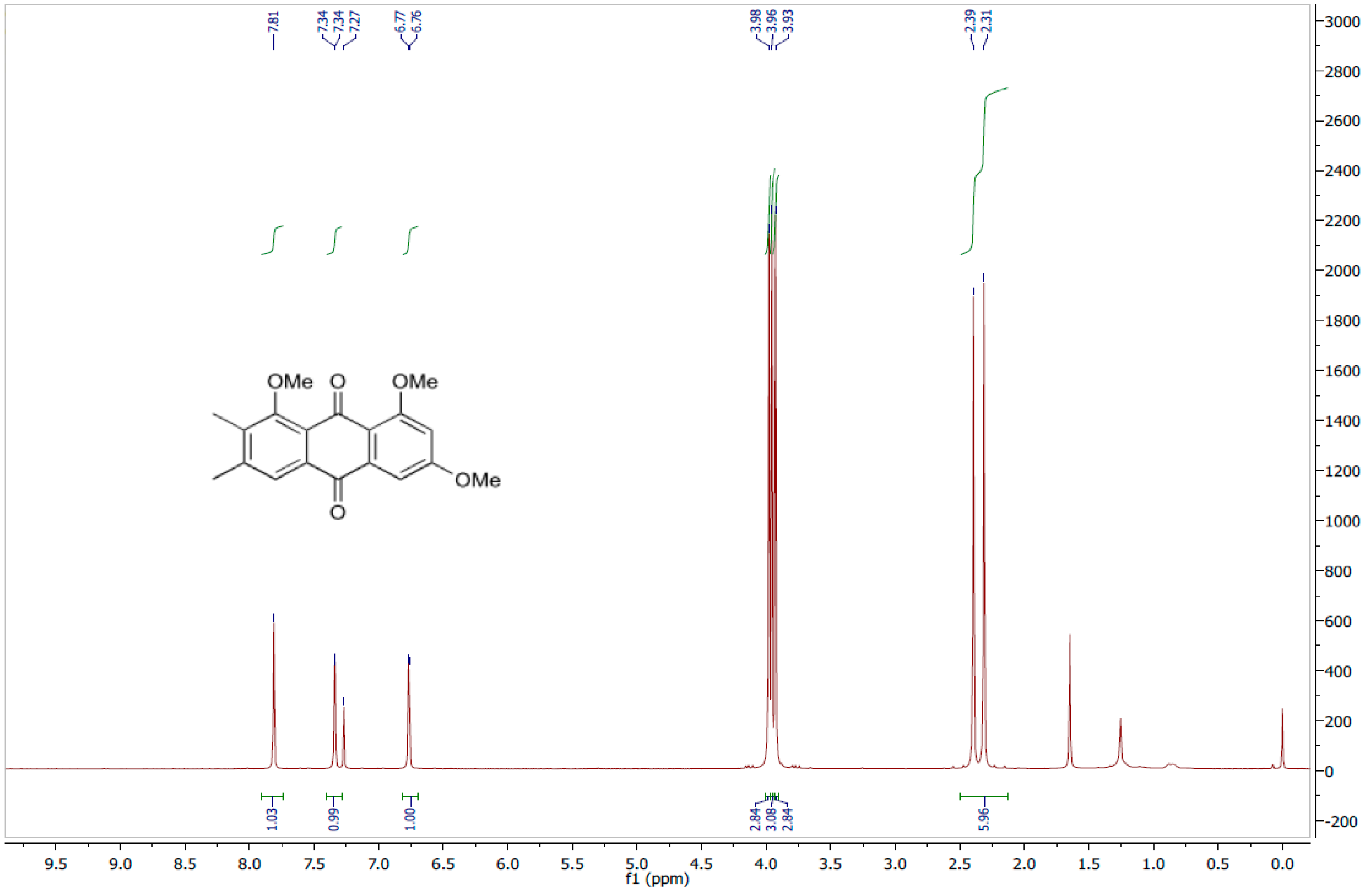

Page S18: Figure S17. ^{1}H NMR spectrum of cichorin F (**3**) (400 MHz, $CDCl_3$).

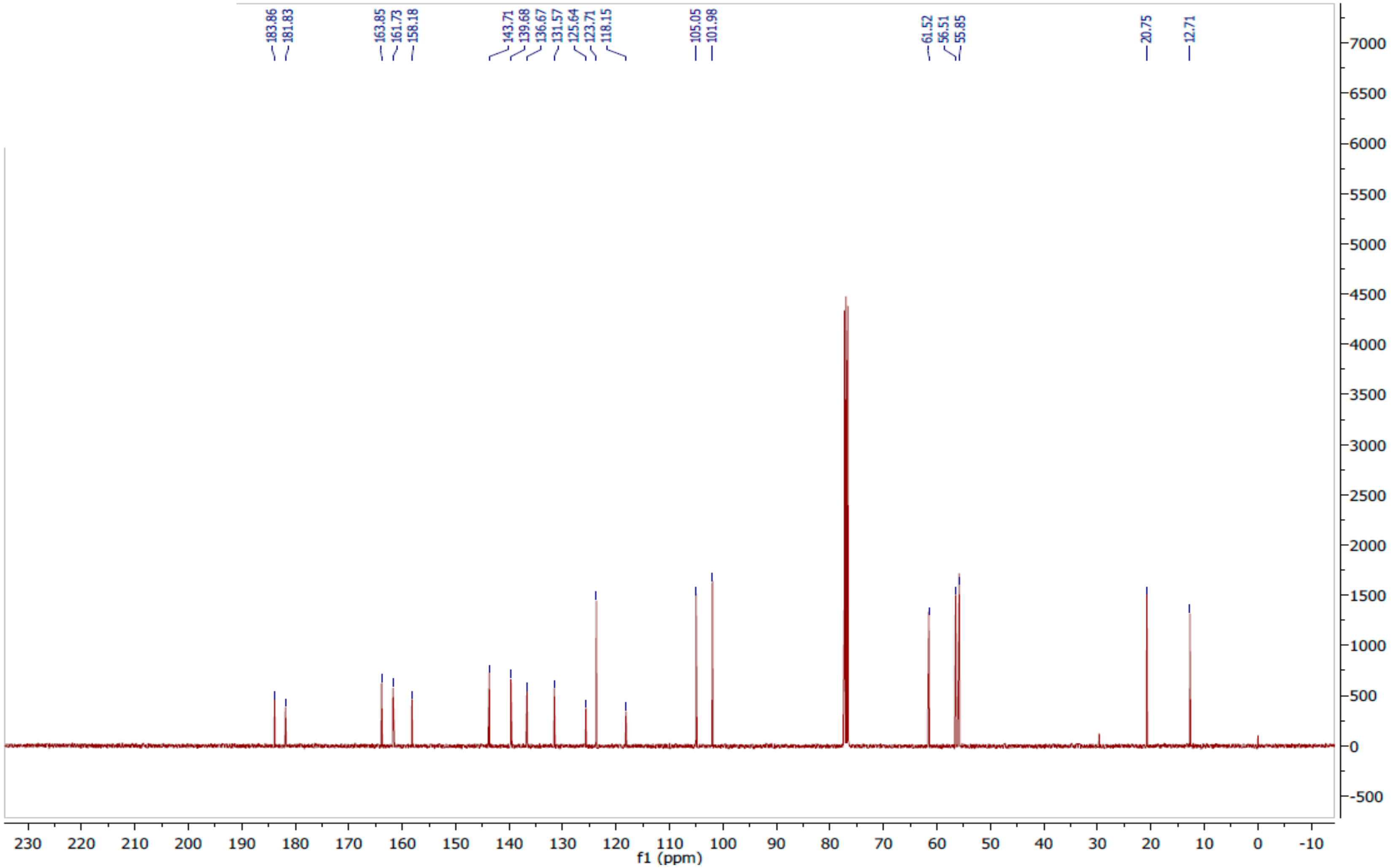

Page S19: Figure S18. ^{13}C NMR spectrum of cichorin F (**3**) (400 MHz, $CDCl_3$).

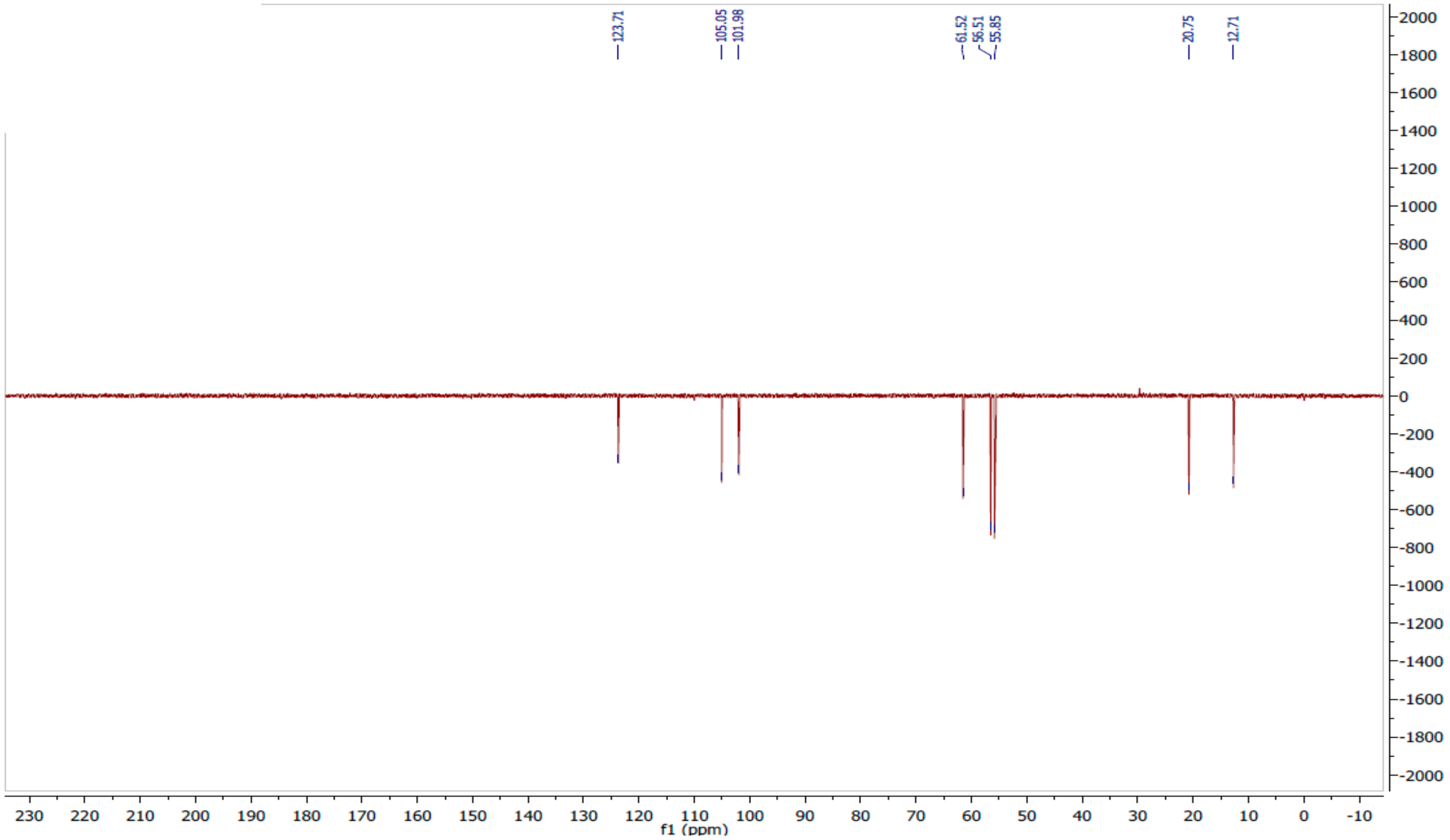

Page S20: Figure S19. DEPT spectrum of cichorin F (**3**) (400 MHz, $CDCl_3$).

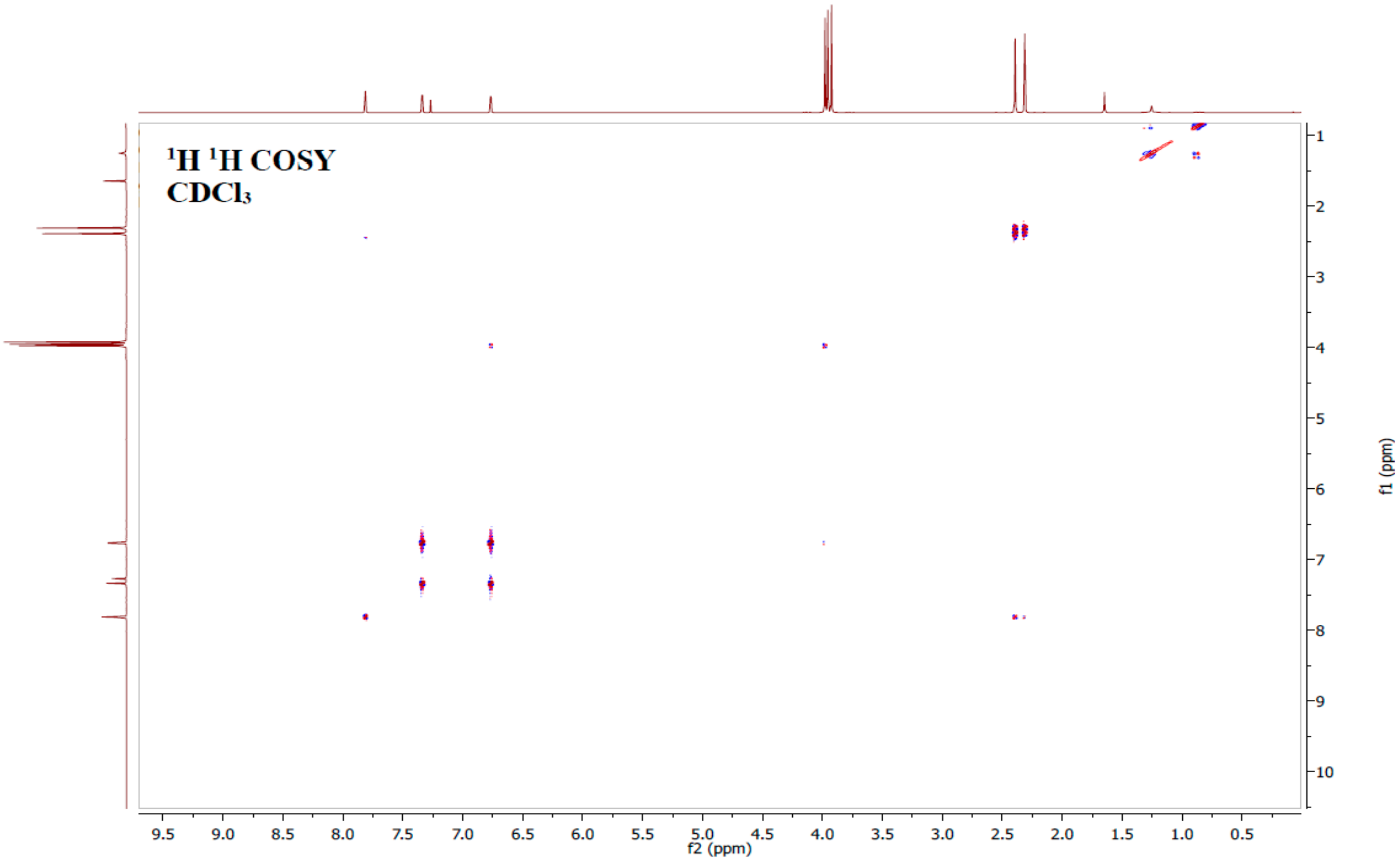

Page S21: Figure S20. COSY spectrum of cichorin F (**3**) (400 MHz, $CDCl_3$).

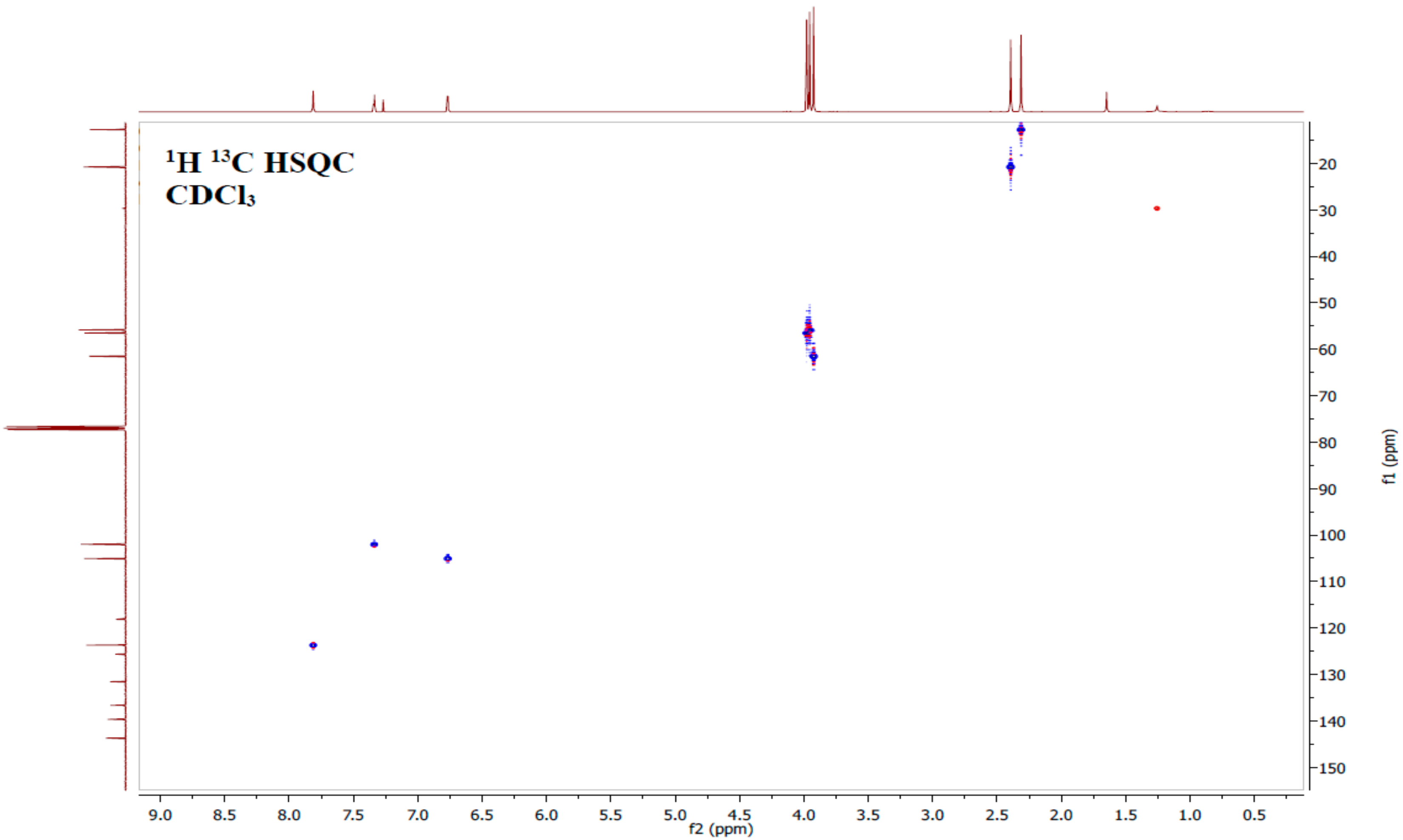

Page S22: Figure S21. HSQC spectrum of cichorin F (**3**) (400 MHz, $CDCl_3$).

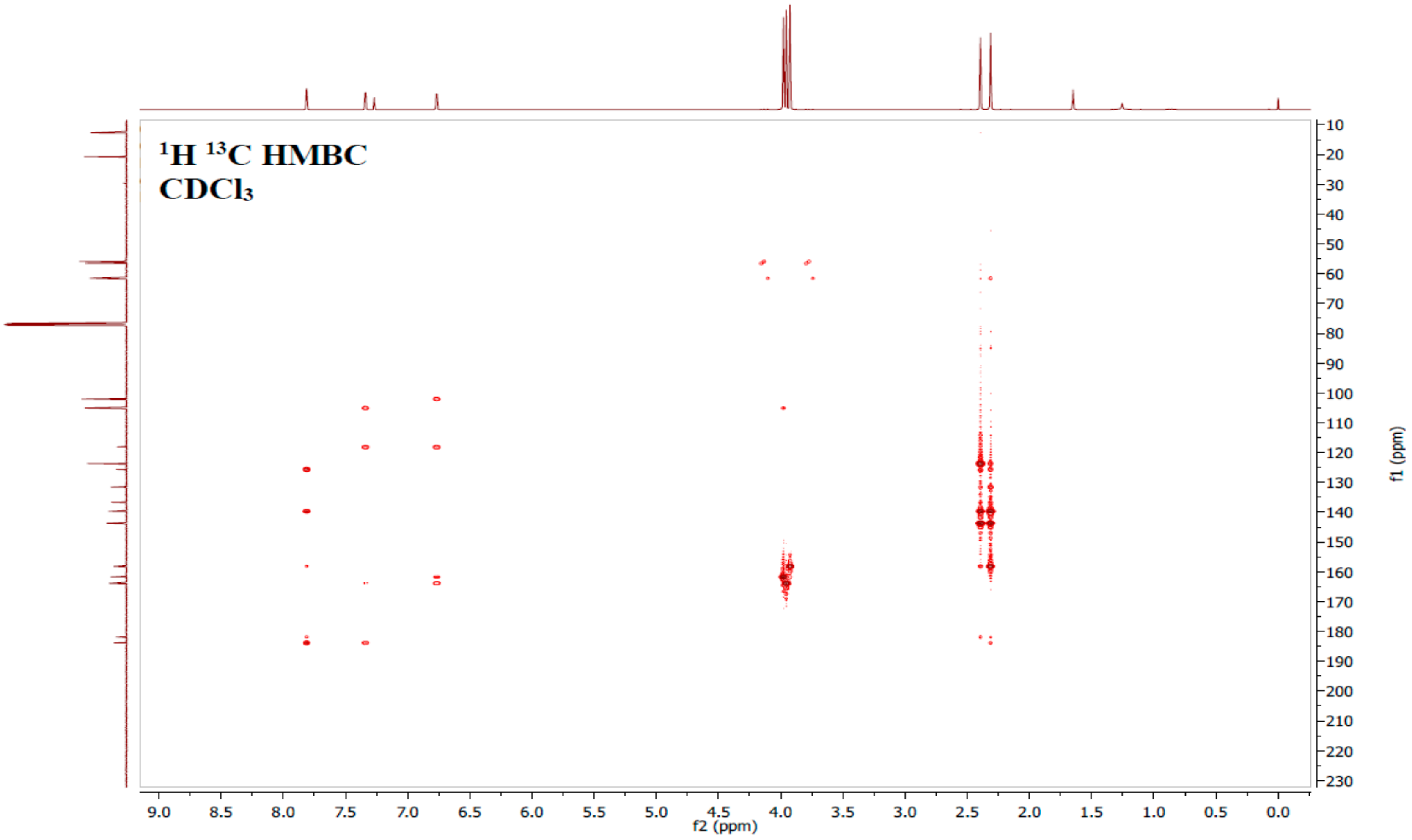

Page S23: Figure S22. HMBC spectrum of cichorin F (**3**) (400 MHz, $CDCl_3$).

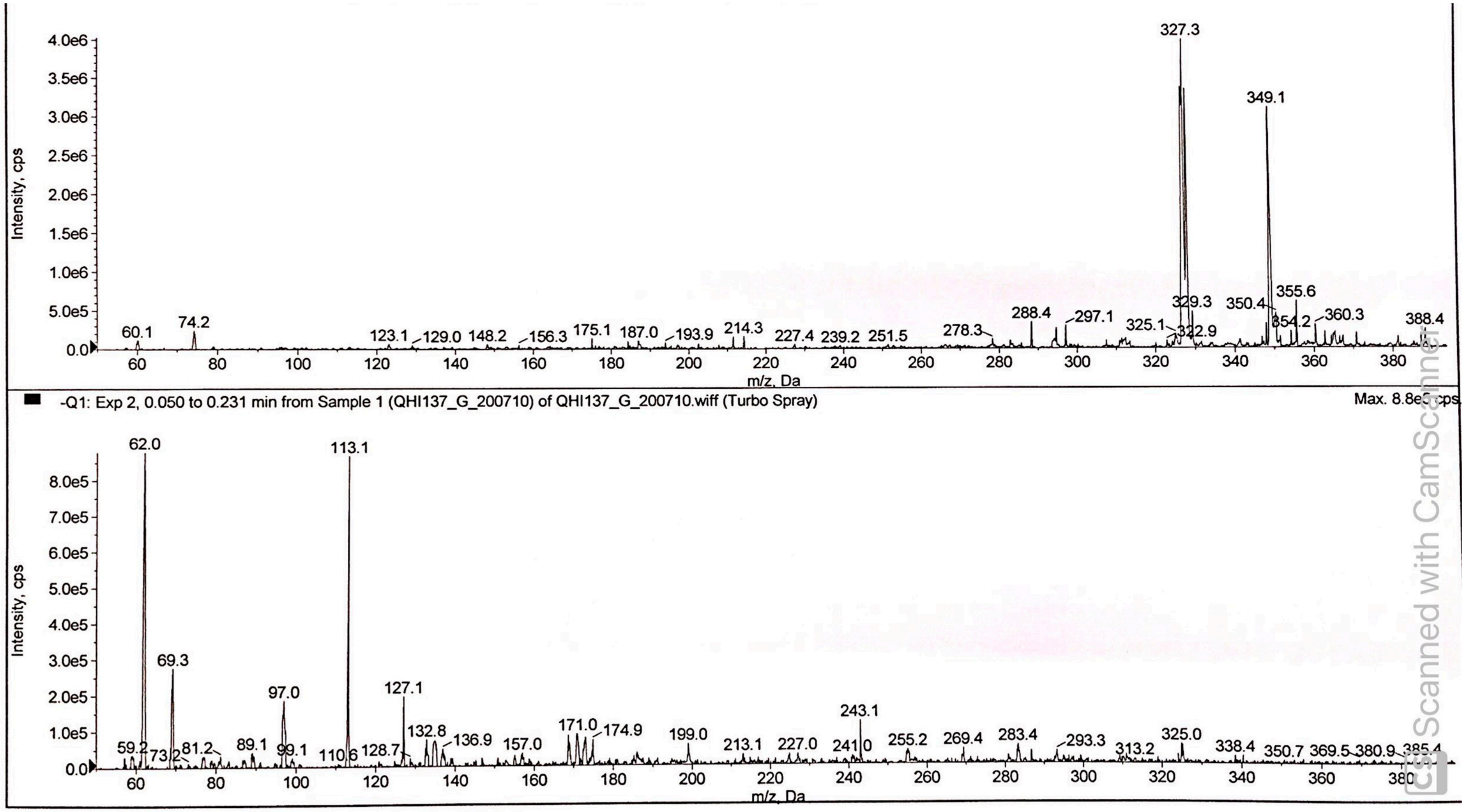

Scanned with CamScanner

Page S24: Figure S23. HRESIMS spectrum of cichorin F (**3**).

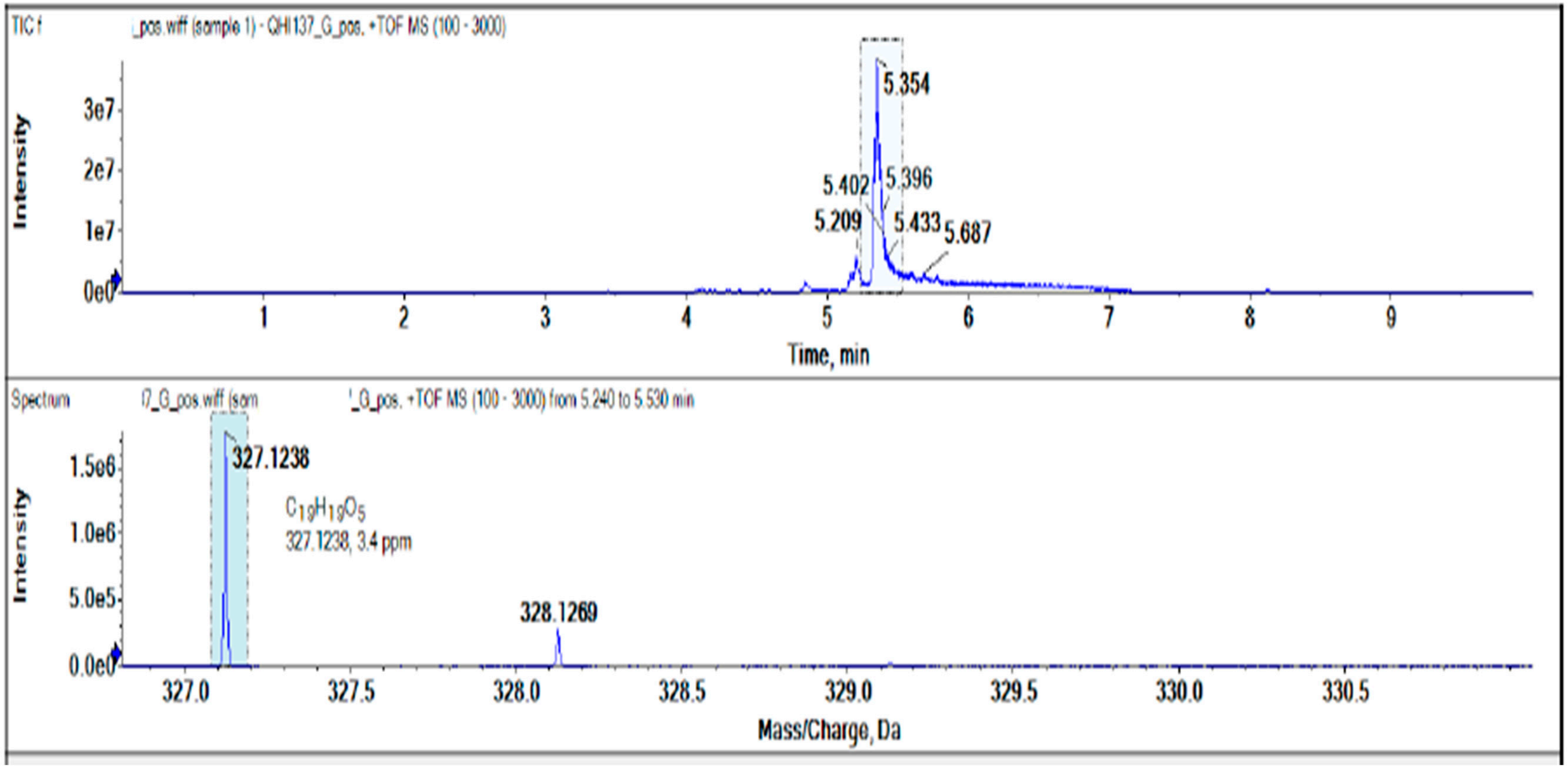

Page S25: Figure S24. HRESIMS spectrum of cichorin F (**3**).
